# Innate immune signatures to a partially-efficacious HIV vaccine predict correlates of HIV-1 infection risk

**DOI:** 10.1371/journal.ppat.1009363

**Published:** 2021-03-15

**Authors:** Erica Andersen-Nissen, Andrew Fiore-Gartland, Lamar Ballweber Fleming, Lindsay N. Carpp, Anneta F. Naidoo, Michael S. Harper, Valentin Voillet, Nicole Grunenberg, Fatima Laher, Craig Innes, Linda-Gail Bekker, James G. Kublin, Ying Huang, Guido Ferrari, Georgia D. Tomaras, Glenda Gray, Peter B. Gilbert, M. Juliana McElrath

**Affiliations:** 1 Vaccine and Infectious Disease Division, Fred Hutchinson Cancer Research Center, Seattle, Washington, United States of America; 2 Cape Town HVTN Immunology Laboratory, Hutchinson Centre Research Institute of South Africa, Cape Town, South Africa; 3 University of Colorado School of Medicine, Aurora, Colorado, United States of America; 4 Perinatal HIV Research Unit, Faculty of Health Sciences, University of the Witwatersrand, Johannesburg, South Africa; 5 The Aurum Institute, Klerksdorp, South Africa; 6 The Desmond Tutu HIV Centre, University of Cape Town, Cape Town, South Africa; 7 Department of Surgery, Immunology, Molecular Genetics and Microbiology, Duke Human Vaccine Institute, Duke University, Durham, North Carolina, United States of America; 8 South African Medical Research Council, Cape Town, South Africa; Vaccine Research Center, UNITED STATES

## Abstract

The pox-protein regimen tested in the RV144 trial is the only vaccine strategy demonstrated to prevent HIV-1 infection. Subsequent analyses identified antibody and cellular immune responses as correlates of risk (CoRs) for HIV infection. Early predictors of these CoRs could provide insight into vaccine-induced protection and guide efforts to enhance vaccine efficacy. Using specimens from a phase 1b trial of the RV144 regimen in HIV-1-uninfected South Africans (HVTN 097), we profiled innate responses to the first ALVAC-HIV immunization. PBMC transcriptional responses peaked 1 day post-vaccination. Type I and II interferon signaling pathways were activated, as were innate pathways critical for adaptive immune priming. We then identified two innate immune transcriptional signatures strongly associated with adaptive immune CoR after completion of the 4-dose regimen. Day 1 signatures were positively associated with antibody-dependent cellular cytotoxicity and phagocytosis activity at Month 6.5. Conversely, a signature present on Days 3 and 7 was inversely associated with Env-specific CD4+ T cell responses at Months 6.5 and 12; rapid resolution of this signature was associated with higher Env-specific CD4+ T-cell responses. These are the first-reported early immune biomarkers of vaccine-induced responses associated with HIV-1 acquisition risk in humans and suggest hypotheses to improve HIV-1 vaccine regimens.

## Author summary

The innate immune response is the body’s initial defense against pathogens and is linked to and shapes the subsequent adaptive immune response, which can confer long-lasting protection. For a vaccine with partial efficacy, such as the RV144 HIV vaccine regimen, identifying early innate responses that are linked with adaptive responses—particularly those for which evidence has accumulated that they might be important for protection—could help a more efficacious version be developed. In the HVTN 097 study, the RV144 prime-boost (ALVAC-HIV and AIDSVAX B/E) vaccine regimen was given to South African participants. We characterized the innate response to the first dose of ALVAC-HIV in these participants and identified gene expression signatures present within the first few days that were associated with antibody and T-cell responses to the full vaccine regimen measured up to 1 year later. As these antibody and T-cell responses have previously been implicated in protection, our findings suggest ways of refining the RV144 regimen and also have broader applications to vaccine development.

## Introduction

Recent estimates of the global impact of HIV/AIDS are a reminder that improved methods of prevention are needed to more effectively address this pandemic. Over 700,000 people died from AIDS-related illnesses in 2018, and over 1.7 million became newly infected [1]. The number of people living with HIV continues to grow, and the economic impact of caring and treating these patients is an immense burden on often fragile health systems [2]. Of the seven preventive HIV-1 vaccine efficacy trials conducted to date [3,4], only the RV144 Thai trial administering the ALVAC-HIV and alum-adjuvanted AIDSVAX B/E prime-boost vaccine regimen has demonstrated efficacy, albeit modest (31%), against HIV acquisition [5]. In RV144, vaccine-induced plasma IgG antibodies recognizing the V1V2 variable region of the HIV envelope glycoprotein (Env) were inversely associated with risk of infection and plasma IgA antibodies to HIV Env were directly associated with risk of infection [6]. In subsequent analyses, the Env-specific CD4+ T cell polyfunctionality (scored by expression of IFN-γ, TNF-α, IL-2, CD40L and IL-4) was also shown as an inverse correlate of risk (CoR) for HIV infection [7]. Post hoc analyses at 12 months of study estimated vaccine efficacy at 60.5% (95% CI 22–80), which then rapidly declined [8]. This decline mirrored the decays in circulating anti-V1V2 antibody [9] and CD4+ T-cell [6] responses, suggesting that vaccine efficacy could be preserved if the magnitude, quality or durability were extended [10]. Vaccine-induced anti-V1V2 IgG antibodies were not broadly neutralizing but did mediate Fc effector functions [11,12]. Vaccine-induced responses were heterogeneous across individuals, suggesting that host factors could account for the variability, and once identified, approaches to increase response rates could be prioritized for future testing.

Systems immunology approaches have helped define innate signatures associated with protective adaptive responses to licensed vaccines in human volunteers [13]. We previously showed that systemic innate responses within the first few days post-vaccination with a non-replicating viral vaccine vector could be used as biomarkers of HIV vaccine immunogenicity [14]. Recent studies have sought to identify adaptive immune transcriptional response signatures of reduced HIV infection risk for the RV144 regimen [15,16], but to date the role of the early innate response to this regimen in shaping adaptive responses in humans has not been defined. Discovery of early biomarkers that predict the immunogenicity of a candidate HIV vaccine could inform efforts to increase protective immunity and lend mechanistic insight into vaccine efficacy, both of which are critical to improve current HIV vaccine regimens. Biomarkers could also identify individuals whose response may be sub-optimal for protection or who may require additional boosting, thereby informing future public health recommendations.

Here we aimed to discover early innate responses associated with CoRs identified during the RV144 trial. We analyzed well-curated, archived samples from HVTN 097, a phase 1b study in which the RV144 vaccine prime-boost regimen was administered to healthy South African volunteers and early specimens were collected [17]. We identify a systemic early IFN response signature to the ALVAC-HIV vector and show that early expression of this signature is associated with higher ADCP activity 2 weeks after the last vaccination. Moreover, rapid resolution of this innate signature correlates with higher magnitude and polyfunctionality of circulating Env-specific CD4+ T-cells 2 weeks and 6 months post-last vaccination. Our results suggest that early strong type I and II IFN responses with rapid resolution could promote development of functional antibody responses and CD4+ T-cell responses to the vaccine antigens.

## Results

### Evaluation of innate immune responses in HVTN 097 participants with a broad range of antibody and T cell responses

**Fig 1:**
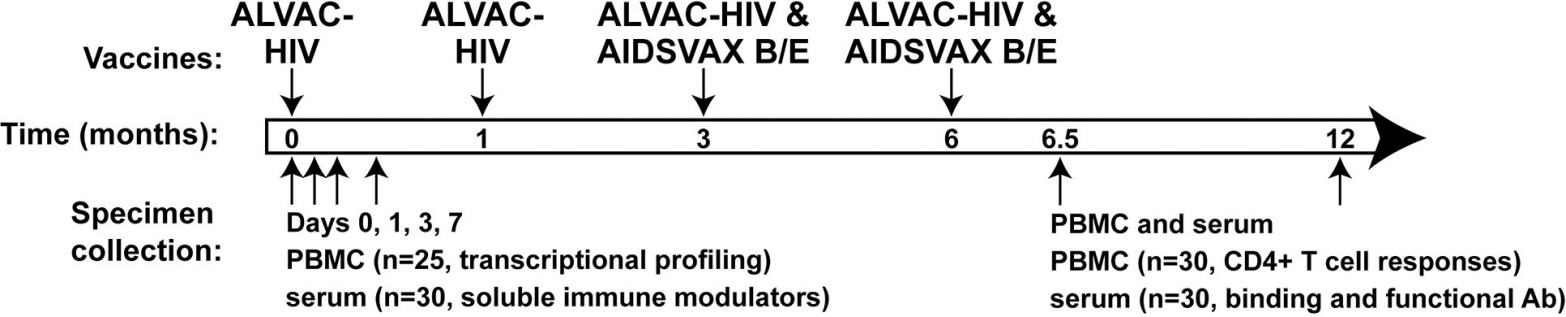
Specimen collection for immune response evaluation in the active groups in the HVTN 097 trial. South African study participants received ALVAC-HIV at Months 0 and 1 followed by ALVAC-HIV and AIDSVAX B/E at Months 3 and 6. PBMC and serum/plasma were collected pre-vaccination and at Days 1, 3 and 7 after the first ALVAC-HIV vaccination for evaluation of innate immune responses and at 2 weeks and 6 months after the fourth vaccination for evaluation of adaptive immune responses.

One hundred healthy, HIV-uninfected South African volunteers at low risk for HIV infection were enrolled in HVTN 097. Volunteers in the two active groups received the RV144 vaccine regimen: 2 doses of ALVAC-HIV (vCP1521), a canarypox vector expressing envelope (clade E), group-specific antigen (Gag) (clade B), and protease (Pro) (clade B), followed by 2 doses of ALVAC-HIV administered with alum-adjuvanted AIDSVAX B/E, a bivalent HIV Env glycoprotein 120 (gp120) [17] (Fig 1). Thirty persons who enrolled in the active groups of the trial volunteered for an intensive specimen collection schedule. Blood was collected immediately before the first ALVAC-HIV administration (M0, defined as “baseline” or “Day 0”) and then 1, 3, and 7 days after the first immunization. Cytokine and chemokine concentrations were measured in the serum. Next-generation RNA sequencing was performed to assess the early peripheral blood mononuclear cell (PBMC) transcriptional responses in 25 of the 30 participants for whom PBMC (previously cryopreserved in an RNA protection reagent) were available at all four timepoints (S1 Table lists demographic information of this subset of participants). These vaccine recipients developed a broad range of humoral and cellular adaptive immune responses measured 6.5 months later (2 weeks post-final ALVAC-HIV/AIDSVAX B/E injection) (S1 Fig, also shown in [17]).

### ALVAC-HIV induces strong type I and II IFN responses in PBMC one day post-vaccination accompanied by an enrichment of monocyte transcripts

**Fig 2:**
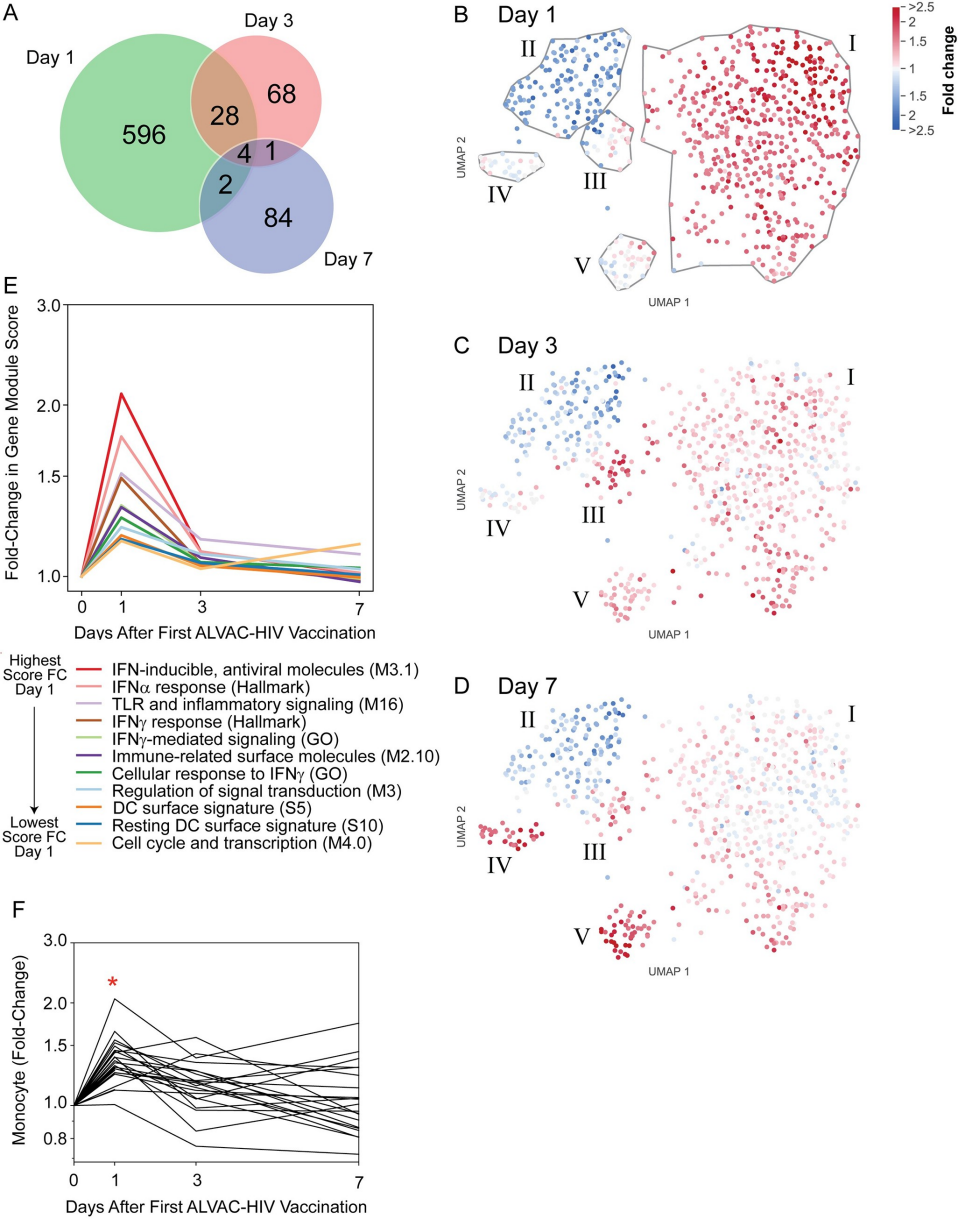
PBMC transcriptional changes after ALVAC-HIV vaccination in 25 vaccine recipients peak at Day 1 and comprise a signature of 11 functional gene modules. **A**) Numbers of differentially-expressed genes (DEGs) on Days 1, 3, and 7 post-vaccination and their overlap. **B-D**) Two-dimensional UMAP embedding of genes (circles) at Day 1 (**B**), Day 3 (**C**), and Day 7 (**D**); genes closer together are more highly correlated across participants and over time. Color intensity indicates log fold-change in expression relative to baseline. Gray lines encircle groups of coordinately expressed genes. **E**) Eleven transcriptional modules were significantly enriched with Day 1 DEGs (FDR-q<0.2). No modules were significantly enriched with Day 3 or Day 7 DEGs. **F**) Cellular enrichment analysis of RNA-seq data using immunoStates [22] showing Day 1 enrichment of monocytes. Each line represents one participant. *significant enrichment versus baseline (FWER < 0.05).

At Day 1 post-ALVAC-HIV immunization, 509 and 121 genes were up- and downregulated, respectively, relative to baseline (Day 0) (S2 Table and S1 Movie). There were fewer differentially-expressed genes (DEGs) by Days 3 and 7 (80 and 79 upregulated and 21 and 12 downregulated, respectively) (S3 Table), suggesting that the innate immune response in PBMC peaked early after vaccination, in accordance with our prior study of a non-replicating HIV vaccine vector [14]. Notably, there was little overlap among the DEGs identified on the different days (Figs 2A and S2 and S3 Tables). Expression of *STAT1*, *OAS1*, and *IRF1* on Days 0, 1 and 3 was confirmed in 21 participants by droplet digital PCR (S3 Fig).

We next conducted gene set enrichment analysis (GSEA) using published transcriptional gene modules (S4 Table). Of the 8,389 tested modules, 11 were significantly enriched with Day 1 DEGs (FDR-q<0.2). No modules were significantly enriched with Day 3 or Day 7 DEGs. The Day 1 response was associated with induction of type I interferon (IFN) and IFN-γ-associated modules. Modules related to dendritic cells (DCs), signal transduction, and Toll-like receptors (TLRs) were also represented (Fig 2E). A mean expression score for each module was computed using all genes in the module, including non-DEGs. All modules increased in expression on Day 1 with subsequent decreases on Days 3 and 7, except the “Cell cycle and transcription (M4.0)” module, which was also higher on Day 7 relative to baseline and may be due to initiation of the adaptive response.

While the modular approach leveraged known patterns of gene expression, we also used an unbiased approach to gene clustering and visualization. DEGs and their patterns of co-expression were embedded in a two-dimensional space using Uniform Manifold Approximation and Projection (UMAP) (Fig 2B–2D and S2 Movie). The genes were clustered to aid further investigation and a heatmap was constructed to show the participant-level variation of fold-change in gene expression for all the DEGs (S4A Fig). Like the DEG-enriched gene modules, many anti-viral and proinflammatory innate response genes were highly upregulated at Day 1 and tended to return to baseline by Day 7 (Cluster I, S4B Fig and S3 Table). Cluster II included genes expressed in T cells and/or NK cells such as *CD69*, *KLRC4* and *KIR3DL1* that were downregulated at Day 1. Cluster III contained a mix of genes that were upregulated on Day 3 and stayed above baseline on Day 7. In addition, the UMAP visualization showed two clusters with a distinct pattern of upregulation at Day 7. Cluster IV contained several immunoglobulin genes, in addition to *MKI67* (Ki67), *TOP2A* and *BIRC5* (Survivin), which are associated with proliferation, likely representing a plasmablast signature [18] as has been reported at Day 7 after immunization with the influenza vaccine (e.g. [19]). Cluster V genes were strongly upregulated at Day 7 and included genes important for erythrocytes, such as *SLC4A1* and *EPB42*, as well as hemoglobin genes, which may be attributed to IFN-γ effects on erythropoiesis [20].

A macaque vaccination study designed to model the RV144 trial suggesting an important role for monocytes in protection [21]. Through a cellular deconvolution analysis [22], we found a significant enrichment of monocyte-associated transcripts in the PBMC at Day 1, with a return to pre-vaccination levels by Day 3 (Fig 2F). CD16+ monocyte-associated transcripts were also enriched at Day 3, suggesting an influx of non-classical monocytes post-vaccination (S5 Fig). The cell enrichment analysis also suggested increases in the plasmacytoid DC population at Day 1, with decreases in T and B cells (S5 Fig).

### Proinflammatory and chemotactic serum factors increase one day after ALVAC-HIV vaccination

**Fig 3:**
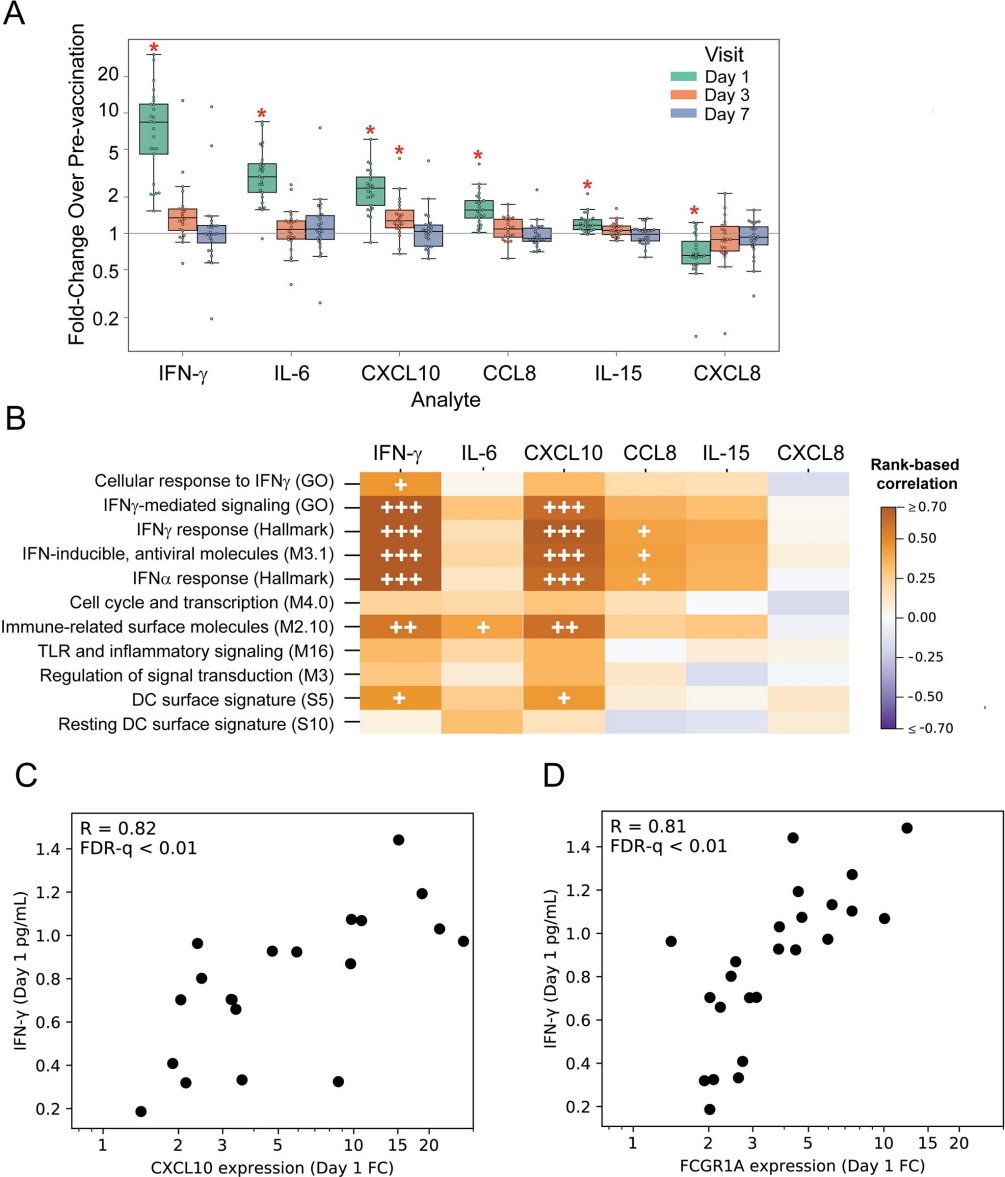
ALVAC-HIV vaccination rapidly induces alterations in serum cytokines and chemokines that are temporally associated with several PBMC gene expression modules (n = 24). **A**) Fold-change serum soluble factors after vaccination. Boxes extend through the interquartile range (IQR), with whiskers extending to the lowest and highest points within 1.5 times the IQR. Asterisks denote significant fold-changes in concentration relative to baseline (FWER-p<0.05, Wilcoxon signed-rank test). **B**) Correlations between fold-change in gene expression at Day 1 for each GSEA gene module with significant Day 1 fold-change in serum factor concentration. FDR-q: <0.2 (+), <0.1 (++), or <0.01 (+++). **C, D**) Scatterplots of Day 1 fold-change in expression of 2 example genes vs. serum IFN-γ concentrations. Each dot represents a single participant; R values represent the strength of the rank-based correlations.

We next used a multiplex platform to measure protein concentrations of 14 soluble serum factors. For the 30 vaccine recipients, changes in serum factors mirrored the gene expression data with most changes observed at Day 1, including significant increases (FWER-p < 0.05) over baseline in the levels of the proinflammatory cytokines IFN-γ and IL-6, myeloid chemotactic factors CXCL10 (IP-10) and CCL8 (MCP-2), and the NK and T cell regulatory factor IL-15 (Fig 3A, mean serum cytokine concentrations for all analytes listed by time point in S5 Table). CXCL10 was the only factor measured that showed higher levels maintained through Day 3. In contrast, CXCL8 (IL-8), important for neutrophil recruitment, was the only factor measured for which the levels decreased after ALVAC-HIV vaccination. Among the analytes that were increased at Day 1, strong correlations were observed among CCL8, IFN-γ and CXCL10 (all R > 0.68, FWER-p < 0.05), demonstrating a coordinated vaccine response (S6 Fig).

To identify whether fold-changes in Day 1 PBMC gene expression were associated with the fold-changes in serum cytokine/chemokine levels, we evaluated correlations between the 11 signature gene modules and each of the cytokines with a significant change at Day 1 (Fig 3B). The strongest correlations were with four IFN-related modules (all FDR-q<0.2). There were strong positive correlations (R>0.75) between CCL8 and three of the IFN-related modules and between IFN-γ and the two of the IFN-related modules (all FDR-q<0.01). Also, several moderate correlations (R>0.50) were observed among the IFN-related modules and CXCL10, CCL8, and IL-15. Significant correlations of cytokine fold-change with activity scores of the 11 modules are listed in S5 Table. To identify individual genes that were potentially driving these correlations, we evaluated the association of each DEG with each of the six cytokines, testing for correlated fold-changes at Day 1. There were 406 genes that were correlated with the concentration of at least one cytokine (FDR-q < 0.2). The strongest correlations were among IFN-related genes and serum IFN-γ concentration- two examples are shown in Fig 3C and 3D. Serum IFN-γ was highly correlated with upregulation of the *CXCL10* (IP-10) gene (Rho = 0.82; FWER-p = 0.004) and with upregulation of the *FCGR1A* gene (Rho = 0.81; FWER-p = 0.008), both of which demonstrate a coordinated anti-viral response and support enrichment and activation of circulating myeloid cells early after vaccination.

As a control, we investigated whether early changes in gene expression profiles or serum cytokines could be detected in participants who volunteered for the intensive innate immune sampling schedule and were randomized to the placebo group. Transcriptional profiling of placebo recipients showed no DEGs (n = 4; FDR-q<0.2 and absolute FC>1.5) (S7 Fig, panel A). Similarly, no significant changes in serum cytokine levels were observed post-vaccination (n = 7; S7 Fig, panel B). In addition, one active group of the HVTN 097 trial included administration of tetanus vaccine one month prior to the first ALVAC-HIV vaccination [17]. We compared baseline module scores and serum cytokine levels of participants who had or had not received the tetanus vaccine one month earlier and did not find differences, indicating that any systemic innate response to the tetanus vaccine had resolved within the month preceding the first ALVAC-HIV vaccination (i.e., by Day 0) (S8 Fig).

### Day 1 serum cytokine responses are associated with Env-specific IgG binding antibody levels

We next sought to identify serum cytokine and gene modules induced by vaccination that were associated with adaptive immune responses previously identified as CoRs or inverse CoRs in the RV144 trial or in non-human primate studies [6,7,9,11,21,23–27]. To this end, we assessed the correlation of the fold-change in serum cytokines or PBMC gene expression modules with binding antibody, CD4+ T cell, antibody-dependent cell-mediated cytotoxicity (ADCC) and antibody-dependent cellular phagocytosis (ADCP) (from [17]; also shown in S1 Fig) at Months 6.5 and 12.

The Day 1 fold-change in IFN-γ, IL-6, CCL8 and IL-15 were each significantly associated with vaccine-matched Env gp120 and V1V2-specific IgG binding antibody levels measured at month 6.5 (FDR-q<0.05, R = 0.45–0.59); a summary score quantifying the response to a breadth of non-vaccine matched gp120 antigens (AUC-MB) was similarly associated (S9A Fig). The gp120-specific IgG responses also remained significantly associated at Month 12 (FDR-q<0.2). There were no significant associations (FDR-q>0.2) of the innate response signature gene modules with any Env-specific IgG or IgA binding antibody responses, although there were weak positive correlations that were consistent with the positive associations with serum cytokines (S9B and S9C Fig and S5 Table). We also evaluated the correlations of individual DEGs with binding antibody responses (IgG BAMA: S3 Movie; IgA BAMA: S4 Movie); only *PML (TRIM19)*, an antiviral factor that can restrict HIV-1 infection [28], was correlated with ALVAC-matched (92TH023) Env-specific IgG measured at Month 6.5 (R = 0.78; FDR-q = 0.07; S5 Table).

### Day 1 serum induction of CCL8 and IFN-γ are associated with Month 6.5 Env-specific ADCC and ADCP; IFN-related gene expression at Day 1 is strongly associated with ADCP

**Fig 4:**
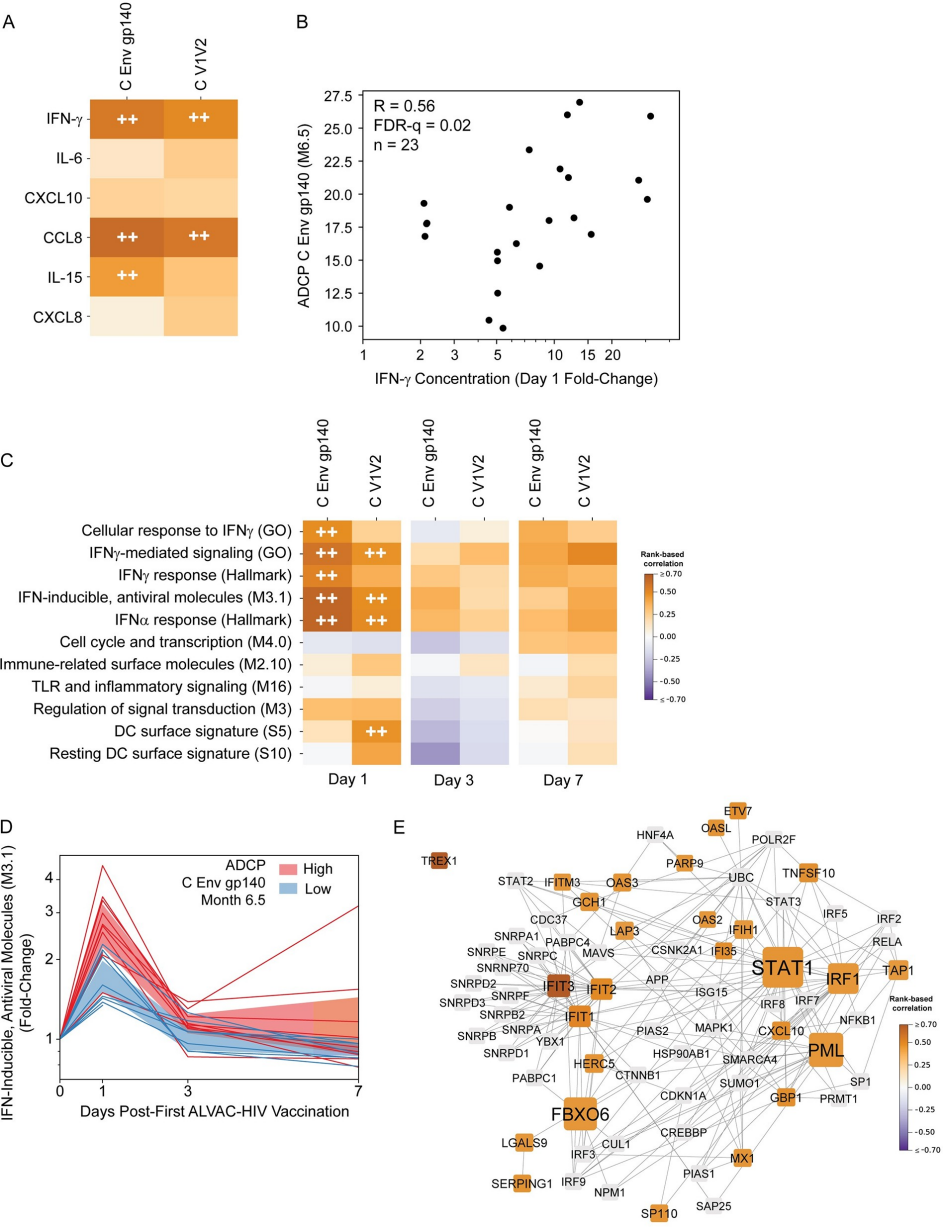
Strong associations between Day 1 changes in gene expression and Month 6.5 Env-specific antibody-dependent cellular phagocytosis (ADCP) responses. **A**) Heatmaps represent the strength of rank-based correlations of fold-change in serum cytokine concentrations with Month 6.5 ADCP responses. HIV-1 Env ADCP antigens are shown in columns. For detailed antigen names, see Materials and Methods. FDR-q <0.1 (++). **B**) Scatterplot of Day 1 fold-change of serum IFN-γ concentration vs. Month 6.5 ADCP C Env gp140 activity. Each dot represents a single participant response; R values in the upper left of the panel represent the strength of the rank-based correlations. **C**) Heatmaps represent the strength of rank-based correlations of fold-change in gene module scores with Month 6.5 ADCP responses. HIV-1 Env antigens are shown in columns. For detailed antigen names, see Materials and Methods. FDR-q <0.1 (++). **D**) “IFN-inducible, antiviral molecules (M3.1)” average module scores at Days 1, 3, and 7. Each line represents the module score for 1 participant. Individual trajectories are colored by Month 6.5 median-split ADCP C Env gp140 activity (blue = low and red = high); shaded region shows 95% CI of mean. **E**) Protein-protein interaction (PPI) network generated from DEGs whose Day 1 log_2_ fold-change showed a significant correlation with Month 6.5 C Env gp140 ADCP responses and that were also present in one of the five gene modules whose Day 1 log_2_ fold-change correlated significantly with Month 6.5 C Env gp140 ADCP responses. Edges represent protein-protein interactions. Nodes are sized according to degree; only nodes that had degree ≥3 in the original network generated from all the genes from the above modules are shown. Nodes are colored according to correlation of Day 1 log_2_ fold-change with C Env gp140 ADCP response magnitude at Month 6.5; only significant correlations are colored. Nodes with non-significant correlations are shown in gray.

Multiple lines of evidence suggest that antibody functions could have contributed to the observed protection in the RV144 trial [6,11] and in other HIV/SHIV vaccine studies [27,29]. Day 1 fold change in serum CCL8 and IFN-γ was significantly associated with Month 6.5 ADCC activity against target cells coated with gp120 protein matching the vector insert (R = 0.56, FDR-q = 0.002 for both; S5 Table); however, the expression of signature gene modules or individual DEGs was not (FDR-q>0.2; S5 Table and S5 Movie). Serum induction of IFN-γ and CCL8 was also associated with both clade C Env gp140 and V1V2-coated bead ADCP activity (R = 0.5–0.62, FDR-q<0.05); serum induction of IL-15 was significantly associated only with Env gp140 ADCP activity (R = 0.44, FDR-q = 0.086) (Fig 4A and 4B).

In addition, associations were observed between innate signature gene module expression with Month 6.5 ADCP activity, which was associated with protection in another HIV vaccine trial using a viral vector [29]. Specifically, moderate positive associations were observed with Day 1 expression of 6 modules: “Cellular response to IFNγ (GO)”, “IFNγ-mediated signaling (GO)”, “IFNγ response (Hallmark)”, “IFN-inducible, antiviral molecules (M3.1)”, “IFNα response (Hallmark)”, and “DC surface signature (S5)” (Fig 4C and S5 Table). Similar to above, we evaluated the correlations of each individual DEG with ADCP responses and visualized the strength of the correlations using the UMAP gene embedding (Env V1V2: S6 Movie, Env gp140: S7 Movie); fold-change of 38 DEGs were associated with V1V2 ADCP activity and 60 DEGs with Env gp140 (S5 Table). Among the most strongly associated genes were several members of the IFN-related signature modules, including IFIT3 (Rho = 0.75, FDR-q = 0.08) a downstream component of the IFN-induced antiviral response [30].

To gain insight into gene pathways driving the association of the early transcriptional response with the subsequent ADCP response, we generated a protein-protein interaction network (shown in Fig 4E) from DEGs in the five modules whose Day 1 fold-change correlated significantly with Month 6.5 ADCP gp140 response (Fig 4A). Many of the network’s hubs were interferon-induced antiviral proteins, including IFIT1, IFIT2, and IFIT3 (interferon-induced proteins with tetratricopeptide repeats), which bind RNA and inhibit viral translation and replication [31]; and PML, a component of PML nuclear bodies, which have multiple antiviral roles [32]. Other hubs were the transcription factors STAT1 and IRF1, which cooperatively upregulate many interferon-stimulated genes to invoke an antiviral state [33,34]. Thus, the network emphasizes the strong antiviral response associations.

### A sustained innate gene signature is associated with lower CD4+ T-cell responses

**Fig 5:**
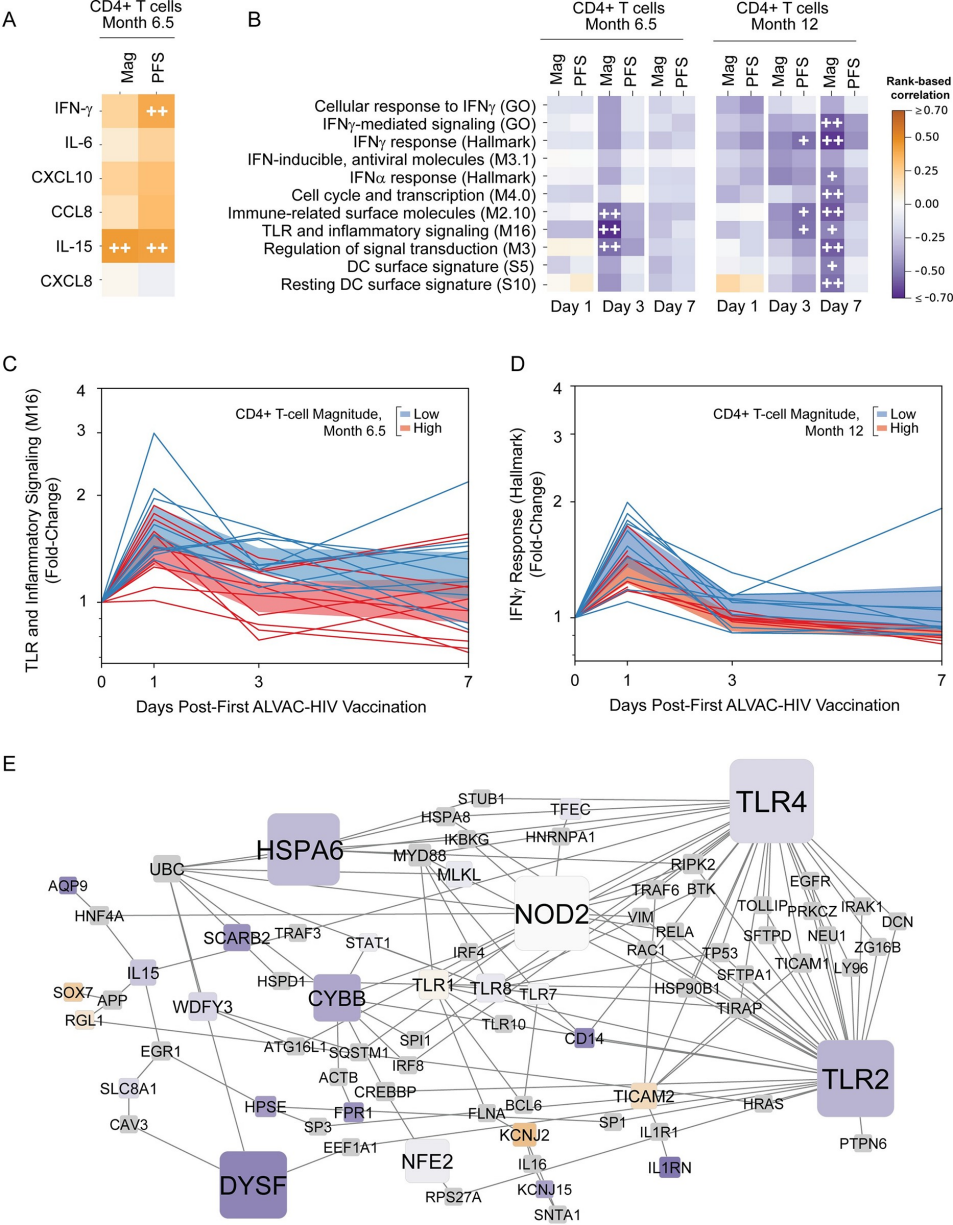
Rapid resolution of the early innate response is associated with higher CD4+ T-cell responses to HIV-1 Env at Months 6.5 and 12. **A**) Heatmaps representing correlations between Month 6.5 Env-specific CD4+ T-cell magnitude (“Mag”) or polyfunctionality score (“PFS”) and Day 1 cytokine fold-change compared to baseline. FDR-q< 0.2 (+) or < 0.1 (++). **B**) Heatmaps representing correlations between average module scores and Month 12 Env-specific CD4+ T-cell Mag and PBS. FDR-q< 0.2 (+) or < 0.1 (++). **C, D**) “TLR and inflammatory signaling (M16)” (**C**) and “IFNγ response (Hallmark)” (**D**) module scores at Days 1, 3, and 7. Each line represents the module score for 1 participant. Individual trajectories colored by Month 6.5 median-split Env-specific CD4+ T-cell response magnitude (blue = low and red = high CD4+ T-cell response); shaded region shows 95% CI of mean. **E**) Protein-protein interaction subnetwork generated from DEGs present in the “Immune-related surface molecules (M2.10)”, “TLR and inflammatory signaling (M16)”, and “Regulation of signal transduction (M3)” modules. Edges represent protein-protein interactions. Nodes are sized according to degree; only nodes that had degree ≥2 in the original network generated from all the genes from the above modules are shown. Nodes are colored according to correlation of Day 3 log fold-change with CD4+ T-cell response magnitude at Month 6.5 (same key as in **A**). Non-DEGs are shown in gray.

Three days after the first ALVAC-HIV administration, the expression levels of the signature gene modules had returned to baseline for many participants, however heterogeneity in expression remained. Therefore, we next examined the associations of gene expression at day 3 with the adaptive responses at Month 6.5 and Month 12. We found that the Env-specific CD4+ T-cell responses were associated inversely with expression of the signature gene modules at Day 3, particularly fold-change in expression of “Immune-related surface molecules (M2.10)” (R = -0.53, FDR-q = 0.08), “TLR and inflammatory signaling (M16)” (R = -0.68, FDR-q = 0.011) and “Regulation of signal transduction (M3)” (R = -0.53, FDR-q = 0.08) modules with Month 6.5 CD4+ T-cell response magnitude (Fig 5A and S5 Table). Inverse correlation indicates that participants with higher levels of gene expression tended to have a lower magnitude CD4+ T cell response. A similar pattern was observed with CD4+ T-cell response magnitude at Month 12, which was inversely associated with changes in gene expression at Day 7 for the same three modules, as well as six other modules tested, including IFN-related, DC-related, and other immune-related modules (Fig 5A). The Env-specific CD4+ T-cell polyfunctionality score at Month 12 was also negatively correlated with Day 3 and Day 7 changes in gene expression, although the associations were not as strong. Significant module correlations are presented in S5 Table. Notably, no module expression changes at Day 1 were associated with the subsequent CD4+ T-cell response. However, there was a moderate and significant positive association between the Day 1 fold-change in IL-15 (R = 0.44, FDR-q = 0.14) and IFN-γ (R = 0.40, FDR-q = 0.17) with the magnitude of the CD4+ T-cell response at Month 6.5.

To visualize the kinetics of the significant associations observed, we plotted the average changes in gene expression for the “TLR and inflammatory signaling (M16)” and the “IFNγ response (Hallmark)” modules over time for each participant (Fig 5B and 5C). Remarkably, participants with a high CD4+ T-cell response at Month 6.5 were more likely to have resolved inflammatory signaling by Day 3 (Fig 5B); similarly, participants with a durable and high CD4+ T-cell response at Month 12 tended to resolve the IFNγ response by Day 7. In contrast to the PPI network of Day 1 modules associated with ADCP that represented many canonical type I IFN-induced genes, the PPI network constructed from DEGs in the three modules whose Day 3 log_2_fold-change correlated with Month 6.5 CD4+ T-cell response (Fig 5B) comprised many genes indicative of myeloid cells. Major hubs in this PPI network (Fig 5D) included the innate sensors *TLR2*, *TLR4*, and *NOD2*; other hubs included *CYBB*, a subunit of the NADPH oxidase complex present in phagocytes, and *DYSF*, which is highly expressed in monocytes [35].

We additionally assessed individual DEGs for correlations with the CD4+ T-cell response measured at Months 6.5 and 12 and found 26 unique genes with significant negative correlations (FDR-q<0.2; S5 Table and S8 Movie): Day 3 *S1PR3* (Cluster I, R = -0.76, FDR-q = 0.14) and Day 3/7 *FCGR1CP* (Cluster I, R = -0.86, FDR-q = 0.098 and R = -0.80, FDR-q = 0.14), consistent with the module-based associations. In contrast, a small group of genes in Cluster I were positively associated particularly on Day 3 with the CD4+ T-cell response. This cluster included genes such as *FADD* and the zinc-finger protein *ZNF366* (S8 Movie), the latter of which has been implicated in dendritic cell potentiation of pro-inflammatory Th1 responses by limiting IL-10 production [36]. One gene, *GORAB*, which is involved in protein glycosylation [37], showed particularly high positive associations at Day 1 and Day 3 with the CD4+ T-cell response (S5 Table and S8 Movie). Altogether, these data suggest that rapid resolution of the innate myeloid response may be a biomarker for protective CD4+ T-cell responses weeks to months after the complete vaccination series.

## Discussion

In this study, we identified human innate immune response signatures of ALVAC-HIV vaccination associated with adaptive immune correlates of HIV-1 infection risk in the RV144 Thai trial. Priming with ALVAC-HIV induced an early antiviral response at Day 1 post-vaccination, modifying expression of more than 600 genes in PBMC and inducing pro-inflammatory and chemotactic serum factors. The innate immune response on Day 1 was associated with the induction of binding antibodies, ADCC and ADCP activity measured 6.5 months later, after all 4 vaccinations in the regimen. We additionally found that resolution of innate transcriptional responses by Day 3 or 7 post-vaccination was associated with higher magnitude and more polyfunctional Env-specific CD4+ T-cell responses to the vaccine regimen. Taken together, these data suggest that a robust early response to vaccination as well as the ability to quickly dampen innate immune activation post-vaccination may lead to protective responses against HIV-1.

Previous studies have identified vaccine response signatures of protection from HIV or SIV acquisition. A nonhuman primate (NHP) study modeling the RV144 vaccine regimen identified monocyte and inflammasome transcriptional signatures that associated with decreased risk of SIV_mac251_ acquisition [21]. Only two of the 88 genes included in the monocyte signature and two of the 8 genes in the inflammasome signature identified in that study were differentially expressed in our HVTN 097 participants, which is likely attributed to differences in study design, including sampling time points and/or response differences between species. We did not measure absolute monocyte counts in HVTN 097, however the cell enrichment analysis indicated that ALVAC-HIV may have induced an increase in circulating monocytes at Days 1 and 3 (Figs 2F and S5). In the NHPs, an early increase in monocytes was a correlate of protection from SIV_mac251_ in a DNA prime, ALVAC/protein boost regimen, which was studied in parallel [21]. In a separate collaborative study of RV144 vaccine recipients, we recently reported a late immune response signature associated with reduced risk of HIV-1 acquisition [16]. In that study, PBMC that were collected two weeks after the final ALVAC/AIDSVAX immunization were stimulated with Env peptide pools and transcriptionally profiled. One of the modules identified in the present study, “IFN-γ response (Hallmark)”, also characterized the response in stimulated PBMC and was associated with decreased risk of infection [16]. These studies suggest that both type I and type II IFN signaling pathways merit study in future HIV-1 vaccine trials.

Early immune response predictors of neutralizing antibody titers have been identified for other vaccines [19,38–41]. Since broadly neutralizing antibodies have not been elicited by an HIV vaccine to date, we instead examined associations with IgG binding and antibody-dependent functional responses that correlated with reduced HIV-1 risk. We found that day 1 increases in serum CCL8, a potent monocyte chemoattractant, were associated with both ADCC and ADCP activity. In addition, a higher early IFN response, evident by increased PBMC expression of both type I and type II IFN-related genes and increased serum concentrations of IFN-γ, was associated with increased Env-specific phagocytic activity measured at Month 6.5; a variety of serum cytokine responses were also positively correlated with the levels of binding antibodies at month 6.5 that would be required for increased ADCP. These data are consistent with models of other viral infections that suggest an ideal type I IFN response is early and transient [42,43] and the finding that virus-specific CD4+ T cell responses in a mouse LCMV infection model can be improved by blocking type I IFN signaling [44]. Taken together, our data generate the hypothesis that the ability to quickly dampen immune activation after vaccination may enhance CD4+ T-cell response to the vaccine inserts.

In our pre-specified analysis plan, gene expression correlations were restricted to the clusters discovered by GSEA and not the data-driven clusters identified and display in the UMAPs (Fig 2B–2D), in order to limit the number of comparisons and thereby reduce the likelihood of false discoveries. However, because other groups have identified plasmablast signatures that are associated with subsequent antibody titers after meningococcal [45], yellow fever [46], malaria [47], and influenza [48] vaccination, we note here that the genes we identified in cluster IV represent a plasmablast signature (S3 Table). Consistent with these other studies, a further exploratory analysis indicates that the increase in expression of genes in cluster IV on Day 7 was positively correlated with 92TH023 gp120 (AE) specific IgG measured at Month 6.5 (rho = 0.48, p = 0.02) and Month 12 (rho = 0.66, p = 0.0009).

In addition to identifying a correlate of adaptive responses, our study provides a detailed assessment of the kinetics and character of the human immune response to ALVAC *in vivo*. We observed a pronounced systemic innate response, involving upregulation of early virus stimulated genes (VSG) [42] (e.g. MX1, OAS2, IRF7) and alterations in serum cytokine and chemokine levels, similar to human studies of other viral vaccine vectors [14,46,49]. *In vitro* studies with human cells have shown preferential induction of type I interferon-related genes over interferon γ-related genes [50] and strong induction of IL-1β [51,52], which we did not observe in our participants. The cytokine profile we observed was also distinct from that seen after ALVAC vaccination of rhesus macaques, where strong induction of IL-1β and IL-10 was not accompanied by induction of IFN-γ [21,51]. In contrast, intramuscular injection of mice with ALVAC resulted in pronounced early IFN-γ production by NK cells [53]. These results suggest that *in vitro* and NHP model systems may not recapitulate the strong IFN-γ component of the innate response we observed in humans.

Specimen collection from donors within the first week after vaccination is challenging to implement in a clinical trial setting. Purified PBMC, analyzed here for consistency with previous work [14], may afford better detection of transcriptional responses from low-abundance cell populations such as dendritic cells, since purification removes high-abundance neutrophils. Over time, RNA stabilized whole blood has become the ideal sample for transcriptional analysis due to its ease of collection and minimal cellular manipulation. Less invasive sampling, such as collection of whole blood using a finger prick [54] rather than venipuncture, could permit extensive kinetic profiling of the innate immune response to candidate HIV vaccines, especially in resource-limited settings. However, additional studies are needed to compare transcriptional signatures obtained from PBMC, whole blood, and low volume samples, to ensure validity of direct comparisons and facilitate meta-analyses.

The kinetics of the innate immune response to ALVAC-HIV closely matched that which we previously observed in a trial of another replication-incompetent vector, Merck adenovirus serotype 5 (MRKAd5) HIV [14]. The MRKAd5-HIV regimen failed to show efficacy in the phase 2b Step and Phambili studies [55–57] and post-hoc analyses even supported enhanced infection in some subgroups of participants [58]. Both the MRKAd5-HIV and ALVAC-HIV vectors induced an increase in expression of interferon related genes one day after the first injection. Although we have not yet performed a formal comparison between the innate immune response to MRKAd5-HIV and ALVAC-HIV in the present study, the dominant response signature in both cases is an interferon response although the modules identified differ by vaccine. Further studies that contrast the signaling pathways induced by different vaccine vectors will be necessary to elucidate key targets to improve immunogenicity and eventually efficacy.

The RV144 and HVTN 097 human clinical trials were followed by a phase 2b/3 efficacy trial, HVTN 702, that tested a similar ALVAC prime/protein boost regimen with modified HIV subtype C immunogens in South Africa [59]. This trial was recently unblinded early for efficacy futility [4]; experiments are currently underway to understand if the immune responses in this trial differed from those seen in RV144 and HVTN 097 or whether the lack of efficacy may also be attributed to differences in adjuvant formulation of the protein boost (MF59 vs alum), the characteristics of the study population and/or the local HIV-1 epidemic. Though HVTN 702 did not collect samples within the first week of vaccination, an ongoing HIV-1 vaccine efficacy study of an adenovirus prime and Env gp140 boost regimen (HVTN 705) will provide an opportunity to test whether the early innate response to the viral vector is predictive of adaptive responses and potentially vaccine efficacy. This and other studies will be important for validating the associations we observed between the innate and adaptive responses and determining if and how they may generalize to other vaccine regimens. Finally, for future development of an effective HIV vaccine, it will be critical to understand the impact of early reduction of the innate immune activation on the effectiveness of vaccine-induced immunity to reduce risk for HIV infection.

## Materials and methods

### Ethics statement

The study was approved by the University of the Witwatersrand Human Research Ethics Committee (Klerksdorp, Soweto sites) and by the University of Cape Town Ethics Committee (Cape Town site). The HVTN 097 trial is registered with the U.S. National Institutes of Health Clinical Trials Registry (ClinicalTrials.gov identifier NCT02109354) and the South African National Clinical Trials Registry (SANCTR number: DOH-27-0313-4201). Voluntary written informed consent was received from participants prior to inclusion in the study.

### RNA sequencing

PBMC were isolated from whole blood collected in ACD anticoagulant and sequenced on an Illumina NextSeq500 with all timepoints for each participant run in the same batch. Differentially expressed genes (DEGs) were defined as having a false discovery rate (FDR) Q value <0.2 and an average absolute fold-change >1.5. Gene set enrichment analysis (GSEA) was performed using the identified DEGs at each time point using published blood transcriptional modules (BTMs) [38,60] and the Hallmark, C2, C4, C5, and C7 gene sets from the Molecular Signatures Database (Broad Institute) [61,62].

### Statistics

#### Gene module-based analysis

To assess correlation of adaptive immunological vaccine-induced responses with gene expression we employed an approach using previously described gene modules (8,389 modules). Modules for downstream analyses were identified based on their enrichment for differentially expressed genes (DEGs) following vaccination (i.e. gene set enrichment analysis, GSEA; significance criteria FDR-q< 0.2 and absolute fold-change > 1.5). A score was calculated for each module and for each participant at each time point based on the average normalized expression level of all genes in the module (DEGs and non-DEGs), on the log scale. Multiplicity adjustment was applied across the 11 modules, across the variables within an assay type (e.g. ICS CD4+ magnitude and polyfunctionality score or BAMA antigens), but within each assay, and within each adaptive and innate immune time point pair; IgA and IgG were considered as two distinct assays. Both family-wise error rate (FWER, Holm-Bonferroni) adjusted p-values and false-discovery rate (FDR, Benjamini-Hochberg) adjusted q-values were computed. Significance was defined as correlations having an FDR-q< 0.2 and unadjusted p<0.05. Additional details are in S1 Text.

#### Multiplex cytokine analysis

Changes in serum cytokines were defined as the log fold-change over pre-vaccine levels. Wilcoxon signed rank tests were used to determine significant changes. Multiple comparison adjustment was performed across analytes to control the family-wise error rate (FWER) using the Holm method [63] (two-sided, α = 0.05).

### Construction of protein-protein interaction networks

Protein-protein interaction networks were generated in NetworkAnalyst [64] using the IMEx interactome database [65]. The list of genes uploaded to NetworkAnalyst to generate the networks consisted of all DEGs present in the designated modules. Networks were imported into Cytoscape [66] for further analysis. Node size represents degree in all networks; nodes were filtered by degree to facilitate visualization.

### Cellular deconvolution

Cell type enrichment analysis of PBMC expression data was performed using immunoStates [22].

### Multiplex cytokine assays, binding antibody multiplex assays (BAMA), intracellular cytokine staining (ICS) assays

Analysis of serum cytokines was performed using the Meso Scale Discovery platform (Rockville, MD). Intracellular cytokine staining assays and binding antibody multiplex assays were performed as in [17]. For ICS assays, the magnitude of the response was defined as the percentage of total CD4+ T cells expressing intracellular IFN-γ and/or IL-2 following *ex vivo* stimulation with Env peptides (T-cell responses from [17]; also shown in S1B Fig); the polyfunctionality score was computed based on the expression of IFN-γ, IL-2, TNF-α, IL-4 and CD154 in the ICS assay using the COMPASS method.

### Detailed BAMA, ADCP, and ADCC antigen names (Figs 4, S1 and S9)

“Vector prime insert” = 92TH023 gp120

“AE protein boost” = Clade AE A244 gp120

“B protein boost” = Clade B MN gp120

“B V1V2 immune correlate” = Clade B gp70-Case A V1V2

“AE V1V2 immune correlate” = Clade AE A244 V1V2

“Env gp140 immune correlate” = A1.Con gp140

“Summary score” = AUC-MB

“C V1V2” = 1086 V1V2

“C Env gp140” = 1086 gp140

### ADCC and ADCP assays

Assays were performed as described in Gray et al. [17].

### Quantitative confirmation of RNA-seq data by reverse transcription-droplet digital PCR (RT-ddPCR)

Two-step RT-ddPCR was used to confirm transcriptomic data for selected genes of interest [67]. Additional details are in S1 Text.

## Supporting information

S1 Text Supplementary methods. (PDF)

S1 Fig Adaptive immune responses in HVTN 097 vaccine recipients used for assessing associations with innate responses. All adaptive immune responses were previously reported in [17] and the subset of participants who enrolled in the innate sampling schedule are shown here for convenience. Responses at Month 6.5 are shown in red; responses at Month 12 are shown in blue. **A**) Binding antibody responses. IgG (left panel) and IgA (right panel) binding antibody levels to HIV Env proteins as measured in a multiplex assay. The summary score is the area under the magnitude-breadth curve [68]. **B**) Antibody-dependent cellular cytotoxicity responses were evaluated against AE 92TH023_gD-neg gp120–coated target cells using peripheral blood mononuclear cells from one normal healthy HIV-seronegative donor as the source of effector cells. The y-axis is the area under the curve (AUC) and represents the response magnitude as detected by release of granzyme B into target cells by flow cytometry. **C**) Antibody-dependent cellular phagocytosis responses. HIV-specific ADCP responses were measured by covalently binding HIV-1 antigens to fluorescent beads and incubating the beads in participant serum to enable formation of immune complexes. The complexes were then incubated with THP-1 monocyte-like cells, after which cell fluorescence was detected by flow cytometry. **D**) HIV-1 Env-specific CD4+ T-cell responses. (Left panel) CD4+ T cell responses as measured in the intracellular cytokine staining (ICS) assay, corresponding to the frequency of CD4+ T cells expressing IL-2 and/or IFN-γ in response to *ex vivo* stimulation with 92TH023 Env overlapping peptide pools. (Right panel) CD4+ T cell polyfunctionality scores based on expression of IFN-γ, IL-2, TNF-α, IL-4 and CD154 in the ICS assay calculated using the COMPASS method [7]. For all plots in **A**) through **D**), median lines are shown and boxes indicate the interquartile range (IQR) with whiskers indicating the two most extreme data points within 1.5 times the IQR. All antigen labels are explained further in Materials and Methods.
(DOCX)

S2 Fig Immune system functions in which the DEGs shared between Days 1 and 3 are involved. The 32 DEGs shared between Days 1 and 3 were input into NetworkAnalyst [64] to generate a protein-protein interaction (PPI) network as described in Materials and Methods. “GO Immune System Process” pathways that were significantly enriched (pV ≤ 0.05) for the resultant genes in the network (n = 187) were analyzed using the ClueGO plugin [69] in Cytoscape [66]. All other settings were used at default. The CluePedia plugin [70] was used to display genes from the original PPI network associated with each enriched GO function or with multiple enriched GO functions.
(DOCX)

S3 Fig Correlations of RNAseq data with ddPCR data for (A) *STAT1*, (B) *OAS1*, and (C) *IRF1* on Day 0 (left column), Day 1 (middle column), and Day 3 (right column). The x-axis of each plot shows the trimmed mean of M-values (TMM)-normalized RNAseq counts and the y-axis shows the mean ratio of ddPCR data.
(DOCX)

S4 Fig **A) Heatmap showing log(fold-change) in gene expression over baseline for the 783 DEGs (FDR≤0.2 and |FC|>1.5) in vaccine recipients at Days 1, 3, and 7 post-ALVAC vaccination.** Each row represents one DEG. Each column represents one individual; the order of individuals is random, but consistent for each day. **B**) Table listing genes of interest from the UMAP clusters (interactive UMAP figure showing the Day 1, Day 3, and Day 7 log_2_ fold-change of each DEG at http://sieve.fredhutch.org/viz/VTN097).
(DOCX)

S5 Fig Cellular enrichment analysis of RNA-seq data performed using immunoStates [22]. Each line represents one participant. Cell types with significant enrichment or depletion compared to baseline (defined as an FWER < 0.05) are marked with a red asterisk on the appropriate day post-first ALVAC-HIV vaccination. The y-axis of each plot shows the proportion (percent) of the given cell type. CD16+ monocytes comprise the less-prevalent non-classical and intermediate monocyte populations; it is likely the day 3 increase is due to an increased proportion of intermediate CD16++ monocytes, similar to that observed after influenza vaccination [48].
(DOCX)

S6 Fig Pairwise rank-based correlations among the fold-changes in concentration at Day 1 of the six serum factors that showed significant changes post-vaccination. R values of rank-based correlations are indicated on the upper right half of the grid and color-coded by the strength of the association; scatterplots are shown on the lower left half of the grid.
(DOCX)

S7 Fig **A**) Heatmap showing log (fold-change) over baseline in placebo recipients for the 783 DEGs (FDR≤0.2 and |FC|>1.5) identified in vaccine recipients at Days 1, 3, and 7 post-ALVAC vaccination. Each row represents one DEG. Each column represents one individual; the order of individuals is random, but consistent for each day. Only placebo recipients (n = 4) are shown. **B**) Fold-change in placebo recipients (n = 7) over pre-vaccination level of the six serum factors that showed significant induction or repression after ALVAC-HIV vaccination. Serum cytokine concentrations were measured using the multiplexed MesoScale Discovery platform. Boxes extend through the interquartile range (IQR), with whiskers extending to the lowest and highest points within 1.5 times the IQR. No factors showed significant fold-changes in concentration relative to baseline (FWER-p <0.05, Wilcoxon signed-rank test).
(DOCX)

S8 Fig Similar baseline profiles for participants who did vs did not receive the tetanus vaccine 1 month before the HIV vaccine regimen. **A**) Median log_2_-reads at baseline (with 95% CI) of the 11 transcriptional modules activated at Day 1, for participants who received the tetanus vaccine (orange) and participants who did not receive the tetanus vaccine (green). **B**) Serum concentrations (pg/ml) at baseline for participants who received the tetanus vaccine (orange, including 7 who received placebo and 22 who received ALVAC-HIV) and participants who did not receive the tetanus vaccine (green) for the 6 cytokines with significant changes at Day 1 post-vaccination. Boxes indicate the interquartile range (IQR), with whiskers indicating the two most extreme data points within 1.5 times the IQR.
(DOCX)

S9 Fig Few associations between early changes in gene expression and Env-specific binding antibody responses. A-B) Heatmaps representing the strength of rank-based correlations of fold-change in gene module scores with Months 6.5 and 12 IgG (A) and IgA (B) binding antibody responses. HIV-1 Env antigens are shown in columns (V1V2 IgG “immune correlate” = inverse correlates of risk in RV144; Env gp140 IgA “immune correlate” = direct correlate of risk in RV144). For detailed antigen names, see S1 Text. FDR-q<0.2 (+) or <0.1 (++).
(DOCX)

S1 Table Demographic data from all HVTN 097 participants and from the subset of participants for whom PBMC transcriptional profiling was performed (median and IQR). (DOCX)

S2 Table Numbers of differentially expressed genes relative to baseline on Days 1, 3, and 7 post-first ALVAC-HIV vaccination. (DOCX)

S3 Table Listing of all differentially expressed genes (DEGs) along with module count, modules, and log_2_-fold-change on Day 1, Day 3, and Day 7. (XLSX)

S4 Table References for modular transcriptional Gene Set Enrichment (GSEA). (DOCX)

S5 Table Listing of correlation testing results including: (1) fold-change in serum cytokines vs. adaptive responses, (2) fold-change of the 11 signature gene modules vs. adaptive responses, (3) fold-change in expression of differentially expressed genes vs. adaptive responses, (4) peripheral cell enrichment vs. adaptive responses, (5) fold-change in serum cytokines vs. fold-change in serum cytokines, (6) adaptive responses vs. adaptive responses. The tables contain all results with unadjusted p < 0.05. A summary of serum cytokine/chemokine concentrations and fold-changes is also provided.
(XLSX)

S1 Movie http://sieve.fredhutch.org/viz/VTN097 Interactive volcano plot showing differentially expressed genes at Days 1, 3, and 7 post-first ALVAC-HIV vaccination. Red circles represent genes with Q-value < 0.2 and fold-change > 1.5.
(HTML)

S2 Movie http://sieve.fredhutch.org/viz/VTN097 Interactive UMAP figure showing the Day 1, Day 3, and Day 7 log2 fold-change of each DEG. (HTML)

S3 Movie http://sieve.fredhutch.org/viz/VTN097 Interactive UMAP figure showing the correlation of Day 1, Day 3, and Day 7 log2 fold-change of each DEG with Month 6.5 (top panels) or Month 12 (bottom panels) IgG binding antibody (BAMA) activity as measured by AUC-MB. (HTML)

S4 Movie http://sieve.fredhutch.org/viz/VTN097 Interactive UMAP figure showing the correlation of Day 1, Day 3, and Day 7 log2 fold-change of each DEG with Month 6.5 (top panels) or Month 12 (bottom panels) IgA binding antibody (BAMA) activity as measured by AUC-MB. (HTML)

S5 Movie http://sieve.fredhutch.org/viz/VTN097 Interactive UMAP figure showing the correlation of Day 1, Day 3, and Day 7 log2 fold-change of each DEG with Month 6.5 antibody-dependent cellular cytotoxicity (ADCC) activity. (HTML)

S6 Movie http://sieve.fredhutch.org/viz/VTN097 Interactive UMAP figure showing the correlation of Day 1, Day 3, and Day 7 log2 fold-change of each DEG with Month 6.5 V1V2-targeted antibody-dependent cellular phagocytosis (ADCP) activity. (HTML)

S7 Movie http://sieve.fredhutch.org/viz/VTN097 Interactive UMAP figure showing the correlation of Day 1, Day 3, and Day 7 log2 fold-change of each DEG with Month 6.5 gp140-targeted antibody-dependent cellular phagocytosis (ADCP) activity. (HTML)

S8 Movie http://sieve.fredhutch.org/viz/VTN097 Interactive UMAP figure showing the correlation of Day 1, Day 3, and Day 7 log2 fold-change of each DEG with Month 6.5 (top panels) or Month 12 (bottom panels) CD4+ T-cell magnitude (proportion of cells expressing IL-2 and/or IFN-γ). (HTML)

## References

[1] UNAIDS. Global HIV & AIDS statistics—2019 fact sheet. https://www.unaids.org/en/resources/fact-sheet.

[2] Meyer-Rath G, van Rensburg C, Chiu C, Leuner R, Jamieson L, Cohen S. The per-patient costs of HIV services in South Africa: Systematic review and application in the South African HIV Investment Case. PLoS One 2019;14(2):e0210497. 10.1371/journal.pone.0210497 30807573 PMC6391029

[3] Tomaras GD, Plotkin SA. Complex immune correlates of protection in HIV-1 vaccine efficacy trials. Immunol Rev. 2017;275(1):245–61. 10.1111/imr.12514 28133811 PMC5330182

[4] Slomski A, Leading HIV. Vaccine Trial Stopped for Ineffectiveness. JAMA. 2020;323(12):1124. 10.1001/jama.2020.2813 32207795

[5] Rerks-Ngarm S, Pitisuttithum P, Nitayaphan S, Kaewkungwal J, Chiu J, Paris R, et al. Vaccination with ALVAC and AIDSVAX to prevent HIV-1 infection in Thailand. N Engl J Med. 2009;361(23):2209–20. 10.1056/NEJMoa0908492 19843557

[6] Haynes BF, Gilbert PB, McElrath MJ, Zolla-Pazner S, Tomaras GD, Alam SM, et al. Immune-correlates analysis of an HIV-1 vaccine efficacy trial. N Engl J Med. 2012;366(14):1275–86. 10.1056/NEJMoa1113425 22475592 PMC3371689

[7] Lin L, Finak G, Ushey K, Seshadri C, Hawn TR, Frahm N, et al. COMPASS identifies T-cell subsets correlated with clinical outcomes. Nat Biotechnol. 2015;33(6):610–6. 10.1038/nbt.3187 26006008 PMC4569006

[8] Robb ML, Rerks-Ngarm S, Nitayaphan S, Pitisuttithum P, Kaewkungwal J, Kunasol P, et al. Risk behaviour and time as covariates for efficacy of the HIV vaccine regimen ALVAC-HIV(vCP1521) and AIDSVAX B/E: a post-hoc analysis of the Thai phase 3 efficacy trial RV 144. Lancet Infect Dis. 2012;12(7):531–7. 10.1016/S1473-3099(12)70088-9 22652344 PMC3530398

[9] Yates NL, Liao HX, Fong YI, deCamp A, Vandergrift NA, Williams WT, et al. Vaccine-Induced Env V1-V2 IgG3 Correlates with Lower HIV-1 Infection Risk and Declines Soon After Vaccination. Science Translational Medicine. 2014;6(228). 10.1126/scitranslmed.3007730 24648342 PMC4116665

[10] Lewis GK, DeVico AL, Gallo RC. Antibody persistence and T-cell balance: two key factors confronting HIV vaccine development. Proc Natl Acad Sci U S A. 2014;111(44):15614–21. 10.1073/pnas.1413550111 25349379 PMC4226080

[11] Chung AW, Kumar MP, Arnold KB, Yu WH, Schoen MK, Dunphy LJ, et al. Dissecting Polyclonal Vaccine-Induced Humoral Immunity against HIV Using Systems Serology. Cell. 2015;163(4):988–98. 10.1016/j.cell.2015.10.027 26544943 PMC5490491

[12] Pollara J, Bonsignori M, Moody MA, Liu P, Alam SM, Hwang KK, et al. HIV-1 vaccine-induced C1 and V2 Env-specific antibodies synergize for increased antiviral activities. J Virol. 2014;88(14):7715–26. 10.1128/JVI.00156-14 24807721 PMC4097802

[13] Hagan T, Pulendran B. Will Systems Biology Deliver Its Promise and Contribute to the Development of New or Improved Vaccines? From Data to Understanding through Systems Biology. Cold Spring Harb Perspect Biol. 2018;10(8). 10.1101/cshperspect.a028894 29038113 PMC5902663

[14] Zak DE, Andersen-Nissen E, Peterson ER, Sato A, Hamilton MK, Borgerding J, et al. Merck Ad5/HIV induces broad innate immune activation that predicts CD8(+) T-cell responses but is attenuated by preexisting Ad5 immunity. Proc Natl Acad Sci U S A. 2012;109(50):E3503–12. 10.1073/pnas.1208972109 23151505 PMC3528489

[15] Ehrenberg PK, Shangguan S, Issac B, Alter G, Geretz A, Izumi T, et al. A vaccine-induced gene expression signature correlates with protection against SIV and HIV in multiple trials. Sci Transl Med. 2019;11(507). 10.1126/scitranslmed.aaw4236 31462510 PMC7383941

[16] Fourati S, Ribeiro SP, Blasco Tavares Pereira Lopes F, Talla A, Lefebvre F, Cameron M, et al. Integrated systems approach defines the antiviral pathways conferring protection by the RV144 HIV vaccine. Nat Commun. 2019;10(1):863. 10.1038/s41467-019-08854-2 30787294 PMC6382801

[17] Gray GE, Huang Y, Grunenberg N, Laher F, Roux S, Andersen-Nissen E, et al. Immune correlates of the Thai RV144 HIV vaccine regimen in South Africa. Sci Transl Med. 2019;11(510). 10.1126/scitranslmed.aax1880 31534016 PMC7199879

[18] Covens K, Verbinnen B, Geukens N, Meyts I, Schuit F, Van Lommel L, et al. Characterization of proposed human B-1 cells reveals pre-plasmablast phenotype. Blood. 2013;121(26):5176–83. 10.1182/blood-2012-12-471953 23613519

[19] Nakaya HI, Wrammert J, Lee EK, Racioppi L, Marie-Kunze S, Haining WN, et al. Systems biology of vaccination for seasonal influenza in humans. Nat Immunol. 2011;12(8):786–95. 10.1038/ni.2067 21743478 PMC3140559

[20] Wang W, Zhao H, Yang Y, Chi Y, Lv X, Zhang L. Interferon-gamma exerts dual functions on human erythropoiesis via interferon regulatory factor 1 signal pathway. Biochem Biophys Res Commun. 2020;521(2):326–32. 10.1016/j.bbrc.2019.10.068 31668371

[21] Vaccari M, Fourati S, Gordon SN, Brown DR, Bissa M, Schifanella L, et al. HIV vaccine candidate activation of hypoxia and the inflammasome in CD14(+) monocytes is associated with a decreased risk of SIVmac251 acquisition. Nat Med. 2018;24(6):847–56. 10.1038/s41591-018-0025-7 29785023 PMC5992093

[22] Vallania F, Tam A, Lofgren S, Schaffert S, Azad TD, Bongen E, et al. Leveraging heterogeneity across multiple datasets increases cell-mixture deconvolution accuracy and reduces biological and technical biases. Nat Commun. 2018;9(1):4735. 10.1038/s41467-018-07242-6 30413720 PMC6226523

[23] Gottardo R, Bailer RT, Korber BT, Gnanakaran S, Phillips J, Shen X, et al. Plasma IgG to linear epitopes in the V2 and V3 regions of HIV-1 gp120 correlate with a reduced risk of infection in the RV144 vaccine efficacy trial. PLoS One. 2013;8(9):e75665. 10.1371/journal.pone.0075665 24086607 PMC3784573

[24] Zolla-Pazner S, deCamp A, Gilbert PB, Williams C, Yates NL, Williams WT, et al. Vaccine-Induced IgG Antibodies to V1V2 Regions of Multiple HIV-1 Subtypes Correlate with Decreased Risk of HIV-1 Infection. Plos One. 2014;9(2). 10.1371/journal.pone.0087572 24504509 PMC3913641

[25] Chung AW, Ghebremichael M, Robinson H, Brown E, Choi I, Lane S, et al. Polyfunctional Fc-effector profiles mediated by IgG subclass selection distinguish RV144 and VAX003 vaccines. Sci Transl Med. 2014;6(228):228ra38. 10.1126/scitranslmed.3007736 24648341

[26] Vaccari M, Gordon SN, Fourati S, Schifanella L, Liyanage NP, Cameron M, et al. Adjuvant-dependent innate and adaptive immune signatures of risk of SIVmac251 acquisition. Nat Med. 2016;22(7):762–70. 10.1038/nm.4105 27239761 PMC5916782

[27] Barouch DH, Stephenson KE, Borducchi EN, Smith K, Stanley K, McNally AG, et al. Protective efficacy of a global HIV-1 mosaic vaccine against heterologous SHIV challenges in rhesus monkeys. Cell. 2013;155(3):531–9. 10.1016/j.cell.2013.09.061 24243013 PMC3846288

[28] Kahle T, Volkmann B, Eissmann K, Herrmann A, Schmitt S, Wittmann S, et al. TRIM19/PML Restricts HIV Infection in a Cell Type-Dependent Manner. Viruses. 2015;8(1). 10.3390/v8010002 PMC4728562 26703718

[29] Neidich SD, Fong Y, Li SS, Geraghty DE, Williamson BD, Young WC, et al. Antibody Fc effector functions and IgG3 associate with decreased HIV-1 risk. J Clin Invest. 2019;129(11):4838–49. 10.1172/JCI126391 31589165 PMC6819135

[30] Johnson B, VanBlargan LA, Xu W, White JP, Shan C, Shi PY, et al. Human IFIT3 Modulates IFIT1 RNA Binding Specificity and Protein Stability. Immunity. 2018;48(3):487–99. e5. 10.1016/j.immuni.2018.01.014 29525521 PMC6251713

[31] Diamond MS, Farzan M. The broad-spectrum antiviral functions of IFIT and IFITM proteins. Nat Rev Immunol. 2013;13(1):46–57. 10.1038/nri3344 23237964 PMC3773942

[32] Scherer M, Stamminger T. Emerging Role of PML Nuclear Bodies in Innate Immune Signaling. J Virol. 2016;90(13):5850–4. 10.1128/JVI.01979-15 27053550 PMC4907236

[33] Abou El Hassan M, Huang K, Eswara MB, Xu Z, Yu T, Aubry A, et al. Properties of STAT1 and IRF1 enhancers and the influence of SNPs. BMC Mol Biol. 2017;18(1):6. 10.1186/s12867-017-0084-1 28274199 PMC5343312

[34] Wang W, Xu L, Su J, Peppelenbosch MP, Pan Q. Transcriptional Regulation of Antiviral Interferon-Stimulated Genes. Trends Microbiol. 2017;25(7):573–84. 10.1016/j.tim.2017.01.001 28139375 PMC7127685

[35] De Luna N, Freixas A, Gallano P, Caselles L, Rojas-Garcia R, Paradas C, et al. Dysferlin expression in monocytes: a source of mRNA for mutation analysis. Neuromuscul Disord. 2007;17(1):69–76. 10.1016/j.nmd.2006.09.006 17070050

[36] Sondergaard JN, van Heeringen SJ, Looman MWG, Tang C, Triantis V, Louche P, et al. Dendritic Cells Actively Limit Interleukin-10 Production Under Inflammatory Conditions via DC-SCRIPT and Dual-Specificity Phosphatase 4. Front Immunol. 2018;9:1420. 10.3389/fimmu.2018.01420 29988341 PMC6023963

[37] Witkos TM, Chan WL, Joensuu M, Rhiel M, Pallister E, Thomas-Oates J, et al. GORAB scaffolds COPI at the trans-Golgi for efficient enzyme recycling and correct protein glycosylation. Nat Commun. 2019;10(1):127. 10.1038/s41467-018-08044-6 30631079 PMC6328613

[38] Li S, Rouphael N, Duraisingham S, Romero-Steiner S, Presnell S, Davis C, et al. Molecular signatures of antibody responses derived from a systems biology study of five human vaccines. Nat Immunol. 2014;15(2):195–204. 10.1038/ni.2789 24336226 PMC3946932

[39] Popper SJ, Strouts FR, Lindow JC, Cheng HK, Montoya M, Balmaseda A, et al. Early Transcriptional Responses After Dengue Vaccination Mirror the Response to Natural Infection and Predict Neutralizing Antibody Titers. J Infect Dis. 2018;218(12):1911–21. 10.1093/infdis/jiy434 30010906 PMC6217718

[40] Bucasas KL, Franco LM, Shaw CA, Bray MS, Wells JM, Nino D, et al. Early patterns of gene expression correlate with the humoral immune response to influenza vaccination in humans. J Infect Dis. 2011;203(7):921–9. 10.1093/infdis/jiq156 21357945 PMC3068032

[41] Rechtien A, Richert L, Lorenzo H, Martrus G, Hejblum B, Dahlke C, et al. Systems Vaccinology Identifies an Early Innate Immune Signature as a Correlate of Antibody Responses to the Ebola Vaccine rVSV-ZEBOV. Cell Rep. 2017;20(9):2251–61. 10.1016/j.celrep.2017.08.023 28854372 PMC5583508

[42] Green R, Ireton RC, Gale M Jr. Interferon-stimulated genes: new platforms and computational approaches. Mamm Genome. 2018;29(7–8):593–602. 10.1007/s00335-018-9755-6 29982912

[43] Channappanavar R, Fehr AR, Vijay R, Mack M, Zhao J, Meyerholz DK, et al. Dysregulated Type I Interferon and Inflammatory Monocyte-Macrophage Responses Cause Lethal Pneumonia in SARS-CoV-Infected Mice. Cell Host Microbe. 2016;19(2):181–93. 10.1016/j.chom.2016.01.007 26867177 PMC4752723

[44] Odorizzi PM, Wherry EJ. Immunology. An interferon paradox. Science. 2013;340(6129):155–6. 10.1126/science.1237568 23580520 PMC4886223

[45] O’Connor D, Clutterbuck EA, Thompson AJ, Snape MD, Ramasamy MN, Kelly DF, et al. High-dimensional assessment of B-cell responses to quadrivalent meningococcal conjugate and plain polysaccharide vaccine. Genome Med. 2017;9(1):11. 10.1186/s13073-017-0400-x 28137280 PMC5282650

[46] Querec TD, Akondy RS, Lee EK, Cao W, Nakaya HI, Teuwen D, et al. Systems biology approach predicts immunogenicity of the yellow fever vaccine in humans. Nat Immunol. 2009;10(1):116–25. 10.1038/ni.1688 19029902 PMC4049462

[47] Kazmin D, Nakaya HI, Lee EK, Johnson MJ, van der Most R, van den Berg RA, et al. Systems analysis of protective immune responses to RTS,S malaria vaccination in humans. Proc Natl Acad Sci U S A. 2017;114(9):2425–30. 10.1073/pnas.1621489114 28193898 PMC5338562

[48] Nakaya HI, Hagan T, Duraisingham SS, Lee EK, Kwissa M, Rouphael N, et al. Systems Analysis of Immunity to Influenza Vaccination across Multiple Years and in Diverse Populations Reveals Shared Molecular Signatures. Immunity. 2015;43(6):1186–98. 10.1016/j.immuni.2015.11.012 26682988 PMC4859820

[49] Gaucher D, Therrien R, Kettaf N, Angermann BR, Boucher G, Filali-Mouhim A, et al. Yellow fever vaccine induces integrated multilineage and polyfunctional immune responses. J Exp Med. 2008;205(13):3119–31. 10.1084/jem.20082292 19047440 PMC2605227

[50] Harenberg A, Guillaume F, Ryan EJ, Burdin N, Spada F. Gene profiling analysis of ALVAC infected human monocyte derived dendritic cells. Vaccine. 2008;26(39):5004–13. 10.1016/j.vaccine.2008.07.050 18691624 PMC7115550

[51] Teigler JE, Phogat S, Franchini G, Hirsch VM, Michael NL, Barouch DH. The canarypox virus vector ALVAC induces distinct cytokine responses compared to the vaccinia virus-based vectors MVA and NYVAC in rhesus monkeys. J Virol. 2014;88(3):1809–14. 10.1128/JVI.02386-13 24257612 PMC3911591

[52] Liu F, Niu Q, Fan X, Liu C, Zhang J, Wei Z, et al. Priming and Activation of Inflammasome by Canarypox Virus Vector ALVAC via the cGAS/IFI16-STING-Type I IFN Pathway and AIM2 Sensor. J Immunol. 2017;199(9):3293–305. 10.4049/jimmunol.1700698 28947539 PMC5679316

[53] Ryan EJ, Harenberg A, Burdin N. The Canarypox-virus vaccine vector ALVAC triggers the release of IFN-gamma by Natural Killer(NK) cells enhancing Th1 polarization. Vaccine. 2007;25(17):3380–90. 10.1016/j.vaccine.2006.12.048 17234309 PMC7115637

[54] Rinchai D, Anguiano E, Nguyen P, Chaussabel D. Finger stick blood collection for gene expression profiling and storage of tempus blood RNA tubes. F1000Res. 2016;5:1385. 10.12688/f1000research.8841.2 28357036 PMC5357033

[55] Gray G, Buchbinder S, Duerr A. Overview of STEP and Phambili trial results: two phase IIb test-of-concept studies investigating the efficacy of MRK adenovirus type 5 gag/pol/nef subtype B HIV vaccine. Curr Opin HIV AIDS. 2010;5(5):357–61. 10.1097/COH.0b013e32833d2d2b 20978374 PMC2995949

[56] Buchbinder SP, Mehrotra DV, Duerr A, Fitzgerald DW, Mogg R, Li D, et al. Efficacy assessment of a cell-mediated immunity HIV-1 vaccine(the Step Study): a double-blind, randomised, placebo-controlled, test-of-concept trial. Lancet. 2008;372(9653):1881–93. 10.1016/S0140-6736(08)61591-3 19012954 PMC2721012

[57] Gray GE, Allen M, Moodie Z, Churchyard G, Bekker LG, Nchabeleng M, et al. Safety and efficacy of the HVTN 503/Phambili study of a clade-B-based HIV-1 vaccine in South Africa: a double-blind, randomised, placebo-controlled test-of-concept phase 2b study. Lancet Infect Dis. 2011;11(7):507–15. 10.1016/S1473-3099(11)70098-6 21570355 PMC3417349

[58] Duerr A, Huang Y, Buchbinder S, Coombs RW, Sanchez J, del Rio C, et al. Extended follow-up confirms early vaccine-enhanced risk of HIV acquisition and demonstrates waning effect over time among participants in a randomized trial of recombinant adenovirus HIV vaccine(Step Study). J Infect Dis. 2012;206(2):258–66. 10.1093/infdis/jis342 22561365 PMC3490694

[59] Bekker LG, Moodie Z, Grunenberg N, Laher F, Tomaras GD, Cohen KW, et al. Subtype C ALVAC-HIV and bivalent subtype C gp120/MF59 HIV-1 vaccine in low-risk, HIV-uninfected, South African adults: a phase 1/2 trial. Lancet HIV. 2018;5(7):e366–e78. 10.1016/S2352-3018(18)30071-7 29898870 PMC6028742

[60] Chaussabel D, Quinn C, Shen J, Patel P, Glaser C, Baldwin N, et al. A modular analysis framework for blood genomics studies: application to systemic lupus erythematosus. Immunity. 2008;29(1):150–64. 10.1016/j.immuni.2008.05.012 18631455 PMC2727981

[61] Liberzon A, Birger C, Thorvaldsdottir H, Ghandi M, Mesirov JP, Tamayo P. The Molecular Signatures Database(MSigDB) hallmark gene set collection. Cell Syst. 2015;1(6):417–25. 10.1016/j.cels.2015.12.004 26771021 PMC4707969

[62] Godec J, Tan Y, Liberzon A, Tamayo P, Bhattacharya S, Butte AJ, et al. Compendium of Immune Signatures Identifies Conserved and Species-Specific Biology in Response to Inflammation. Immunity. 2016;44(1):194–206. 10.1016/j.immuni.2015.12.006 26795250 PMC5330663

[63] Holm S. A Simple Sequentially Rejective Multiple Test Procedure. Scand J Stat. 1979;6(2):65–70.

[64] Xia J, Benner MJ, Hancock RE. NetworkAnalyst—integrative approaches for protein-protein interaction network analysis and visual exploration. Nucleic Acids Res. 2014;42(Web Server issue):W167–74. 10.1093/nar/gku443 24861621 PMC4086107

[65] Breuer K, Foroushani AK, Laird MR, Chen C, Sribnaia A, Lo R, et al. InnateDB: systems biology of innate immunity and beyond—recent updates and continuing curation. Nucleic Acids Res. 2013;41(Database issue):D1228–33. 10.1093/nar/gks1147 23180781 PMC3531080

[66] Shannon P, Markiel A, Ozier O, Baliga NS, Wang JT, Ramage D, et al. Cytoscape: a software environment for integrated models of biomolecular interaction networks. Genome Res. 2003;13(11):2498–504. 10.1101/gr.1239303 14597658 PMC403769

[67] Pinheiro LB, Coleman VA, Hindson CM, Herrmann J, Hindson BJ, Bhat S, et al. Evaluation of a droplet digital polymerase chain reaction format for DNA copy number quantification. Anal Chem. 2012;84(2):1003–11. 10.1021/ac202578x 22122760 PMC3260738

[68] Huang Y, Gilbert PB, Montefiori DC, Self SG. Simultaneous Evaluation of the Magnitude and Breadth of a Left and Right Censored Multivariate Response, with Application to HIV Vaccine Development. Stat Biopharm Res. 2009;1(1):81–91. 10.1198/sbr.2009.0008 20072667 PMC2805400

[69] Bindea G, Mlecnik B, Hackl H, Charoentong P, Tosolini M, Kirilovsky A, et al. ClueGO: a Cytoscape plug-in to decipher functionally grouped gene ontology and pathway annotation networks. Bioinformatics. 2009;25(8):1091–3. 10.1093/bioinformatics/btp101 19237447 PMC2666812

[70] Bindea G, Galon J, Mlecnik B. CluePedia Cytoscape plugin: pathway insights using integrated experimental and in silico data. Bioinformatics. 2013;29(5):661–3. 10.1093/bioinformatics/btt019 23325622 PMC3582273

