# Supplementary material for: Innate immune signatures to a partially-efficacious HIV vaccine predict correlates of HIV-1 infection risk
Source: PLoS Pathog. 2021 Mar 15;17(3):e1009363. doi: 10.1371/journal.ppat.1009363 (PMC7959397; doi:10.1371/journal.ppat.1009363)
Supplement: S1 Text — (PDF) [file ppat.1009363.s001.pdf]

Supplementary Methods

HVTN 097 trial regimen

Volunteers in the two active arms received the RV144 vaccine regimen: 2 doses of ALVAC-HIV (vCP1521), a canarypox vector expressing envelope (clade E), group-specific antigen (Gag) (clade B), and protease (Pro) (clade B), followed by 2 doses of ALVAC-HIV administered with alum-adjuvanted AIDSVAX® B/E, a bivalent HIV Env glycoprotein 120 (gp120) *(17)*.

RNA sequencing

PBMC were resuspended in RNAprotect Cell Reagent (Qiagen, Valencia, CA) immediately after isolation according to the manufacturer's instructions. RNA was extracted using the RNeasy Cell Protect protocol (Qiagen) and its integrity assessed with Agilent RNA 6000 Nano kit and Agilent 2200 TapeStation (Agilent Technologies). Total RNA concentrations were determined using the Quant-IT RNA assay (ThermoFisher, Waltham, MA) per the manufacturer's instructions on a Qubit 2.0 Fluorometer (ThermoFisher).

Libraries were constructed using 250 ng of total RNA with a TruSeq Stranded mRNA Sample Preparation kit (Illumina, San Diego, CA), which incorporates poly(A)+RNA selection of mRNA, according to the manufacturer's protocol. Sequencing libraries were quantified by qPCR using the KAPA Library Quantification Kit for Illumina Libraries (KapaBiosystems, Wilmington, MA) and library profiles assessed using the Agilent DNA 1000 kit and Agilent 2200 Tapestation. Libraries were sequenced on an Illumina NextSeq500 instrument using a 150-cycle flow cell version 2.5 chemistry in paired end mode (2x75). Each sample had a read depth of at least 20 million paired end reads per sample. The total number of mean paired-end reads for all visits was 55.4 million (median 33.1 million; range 10.2-298.4 million). The mean Phred score for all visits was 33.2 (median 32.8, range 29.5-35.6).

Raw FASTQ files were uploaded to the Partek Flow version 6 software and assessed for quality and then processed first by filtering for low quality reads and trimming adapter sequences and removing reads aligned to rRNA and tRNA (Bowtie). The remaining high-quality reads were mapped to hg38 build of the human genome using the University of California Santa Cruz genome browser database (*60*) and the STAR - 2.5.3a aligner. Aligned reads were then quantified to the annotation model (Partek E/M) at the gene-level using hg38 GENCODE Release 26 (*61*). Genes with fewer than 20 reads were excluded from further analysis. The remaining raw gene counts were normalized using the trimmed means of the m-value normalization method (*62*) and then offset by 1 to avoid zero counts.
Differential expression at each time point compared to baseline was assessed using normalized counts with the estimated fold-change dispersion model fitted by DESEQ2 (*63*).

Deconvolution of RNA-seq data

The MetaIntegrator R package was used for deconvolution analysis (*22*). For each cell type, a Wilcoxon signed-rank test was used to identify the significant changes in cell frequency over baseline. A Holm-Bonferroni adjustment was applied as correction for multiple testing; a FWER-p < 0.05 is reported as significant.

Multiplex cytokine analysis

Analysis of serum cytokines was performed using the following kits from Meso Scale Discovery (Rockville, MD): MCP-2 Panel – K151MID-2 (MCP-2), Chemokine Panel –

K15047G-2 (Eotaxin, IP-10, MCP-1), Proinflammatory Panel - K15049D-2 (IFN-γ, IL-1β, IL-6, IL-8, IL-12p70, TNF-α, and IL-10), and Cytokine Panel – K15050D-2 (GM-CSF, IL-1α, and IL-15) and run according to the manufacturer's instructions. Data was analyzed using the MSD Discovery Workbench Software (Meso Scale Discovery, Rockville, MD). Changes in serum cytokines were defined as the log fold-change over pre-vaccine levels for several time points post-vaccination. Wilcoxon signed rank tests were used to determine significant changes (two-sided α=0.05). Multiple comparison adjustment was performed across analytes using the Hochberg method (*64*).

Module-based analysis

The primary analysis assessing correlation of adaptive immunological vaccine-induced responses with gene expression was conducted using modules of genes. Module-based analyses preserve statistical power by testing groups of genes together, reducing the number of tests and increasing signal through within-module averaging. Modules can also increase the interpretability of results and have been used extensively for this purpose (*38, 51*). Though *de novo* clustering of genes could have been used to form modules, we pre-specified a strategy that would leverage pre-existing modules (8,389 total modules), many of which have been previously associated with vaccine response. A primary set of modules for downstream analyses were identified based on their enrichment for differentially expressed genes following vaccination (GSEA). The GSEA analysis identified 11 modules that were significantly enriched with Day 1 DEGs (FDR-q< 0.2 and abs-FC > 1.5); no modules were significantly enriched on Days 3 and 7. A score was calculated for each module and for each participant at each time point based on the average normalized expression level of all genes in the module (DEGs and non-DEGs), on the log scale. From these scores, fold-change (FC) over baseline was computed for Days 1, 3 and 7. Both the expression score at baseline and Days 1, 3 and 7, as well as the FC at Days 1, 3 and 7 were assessed as potential correlates of each adaptive response variable using Spearman's rank correlation; rank correlation is robust to outliers, non-Gaussian distributed data and monotonic transformations. Multiplicity adjustment was computed across the 11 modules, across the variables within an assay type (e.g. ICS CD4+ magnitude and polyfunctionality score or BAMA antigens), but within each adaptive and innate immune time point (e.g. RNA sampling Day 1 and ICS sampling Month 6.5). Both family-wise error rate (FWER, Holm-Bonferroni) adjusted p-values and false-discovery rate (FDR, Benjamini-Hochberg) adjusted q-values were computed. Significance was defined as correlations having an FDR-q< 0.2 and unadjusted p<0.05. With this threshold it is estimated that up to 20% of the significant results are expected to be false positives. An exploratory analysis was performed to assess the correlation of each DEG with the adaptive immune measurements.

Gene embedding and clustering for visualization

For the purpose of visualization, genes were embedded and plotted in a two-dimensional space (Fig 2) using the method of Uniform Manifold Approximation and Projection (UMAP) (*65*). A similarity kernel was computed for all pairs of genes based on their Pearson correlation in expression across participants and time points (Days 0, 1, 3, 7). UMAP attempts to embed the genes in a 2-D Euclidean space that preserves these similarities with high-fidelity by placing similar genes closer to one another (parameters: $N_{neighbors}$=50, D_{min}=0.5); information is lost in any embedding or dimensionality reduction, but UMAP has demonstrated an ability to preserve elements of both local and global structure for effective visualization of large datasets. Genes were grouped by hand to aid interpretation. Both the UMAP plot and heatmap represent fold-change on a log-scale with a truncation at ±2.5-fold.

Quantitative confirmation of RNA-seq data by reverse transcription-droplet digital PCR (RT-ddPCR)

cDNA was generated using the High Capacity cDNA Reverse Transcription Kit (Thermo Fisher Scientific) and the ddPCR was carried out using the 2× ddPCR Supermix for Probes (BioRad, Hercules, CA). Reactions were set up as a duplex reaction with 20× 6-carboxyfluorescein (FAM)-labeled target gene-specific qPCR assay (Integrated DNA Technologies, Coralville, IA) and 20× HEX-labeled housekeeping POL2RA gene-specific Taqman gene expression assay (Thermo Fisher Scientific). Each assembled ddPCR reaction mixture was loaded in duplicate and droplets were generated using the AutoDG (Biorad) auto droplet generator using disposable droplet generator cartridges (BioRad). After droplet generation, the plate was sealed and samples amplified to endpoint in 96-well PCR plates on a conventional thermal cycler using the following conditions: denaturation/enzyme activation for 10 min at 95°C, 40 cycles of 30 s denaturation at 94°C, and 60 s annealing/amplification at 60°C, followed by a final 10 min incubation step at 98°C. After PCR, the droplets were read on the QX200 Droplet Reader (BioRad). Analysis of the ddPCR data was performed with QuantaSoft analysis software version 1.4 (BioRad).
