# Supplementary material for: Innate immune signatures to a partially-efficacious HIV vaccine predict correlates of HIV-1 infection risk
Source: PLoS Pathog. 2021 Mar 15;17(3):e1009363. doi: 10.1371/journal.ppat.1009363 (PMC7959397; doi:10.1371/journal.ppat.1009363)
Supplement: S1 Fig — All adaptive immune responses were previously reported in [17] and the subset of participants who enrolled in the innate sampling schedule are shown here for convenience. Responses at Month 6.5 are shown in red; responses at Month 12 are shown in blue. A) Binding antibody responses. IgG (left panel) and IgA (right panel) binding antibody levels to HIV Env proteins as measured in a multiplex assay. The summary score is the area under the magnitude-breadth curve [68]. B) Antibody-dependent cellular cytotoxicity responses were evaluated against AE 92TH023_gD-neg gp120–coated target cells using peripheral blood mononuclear cells from one normal healthy HIV-seronegative donor as the source of effector cells. The y-axis is the area under the curve (AUC) and represents the response magnitude as detected by release of granzyme B into target cells by flow cytometry. C) Antibody-dependent cellular phagocytosis responses. HIV-specific ADCP responses were measured by covalently binding HIV-1 antigens to fluorescent beads and incubating the beads in participant serum to enable formation of immune complexes. The complexes were then incubated with THP-1 monocyte-like cells, after which cell fluorescence was detected by flow cytometry. D) HIV-1 Env-specific CD4+ T-cell responses. (Left panel) CD4+ T cell responses as measured in the intracellular cytokine staining (ICS) assay, corresponding to the frequency of CD4+ T cells expressing IL-2 and/or IFN-γ in response to ex vivo stimulation with 92TH023 Env overlapping peptide pools. (Right panel) CD4+ T cell polyfunctionality scores based on expression of IFN-γ, IL-2, TNF-α, IL-4 and CD154 in the ICS assay calculated using the COMPASS method [7]. For all plots in A) through D), median lines are shown and boxes indicate the interquartile range (IQR) with whiskers indicating the two most extreme data points within 1.5 times the IQR. All antigen labels are explained further in Materials and Methods. (DOCX) [file ppat.1009363.s002.docx]

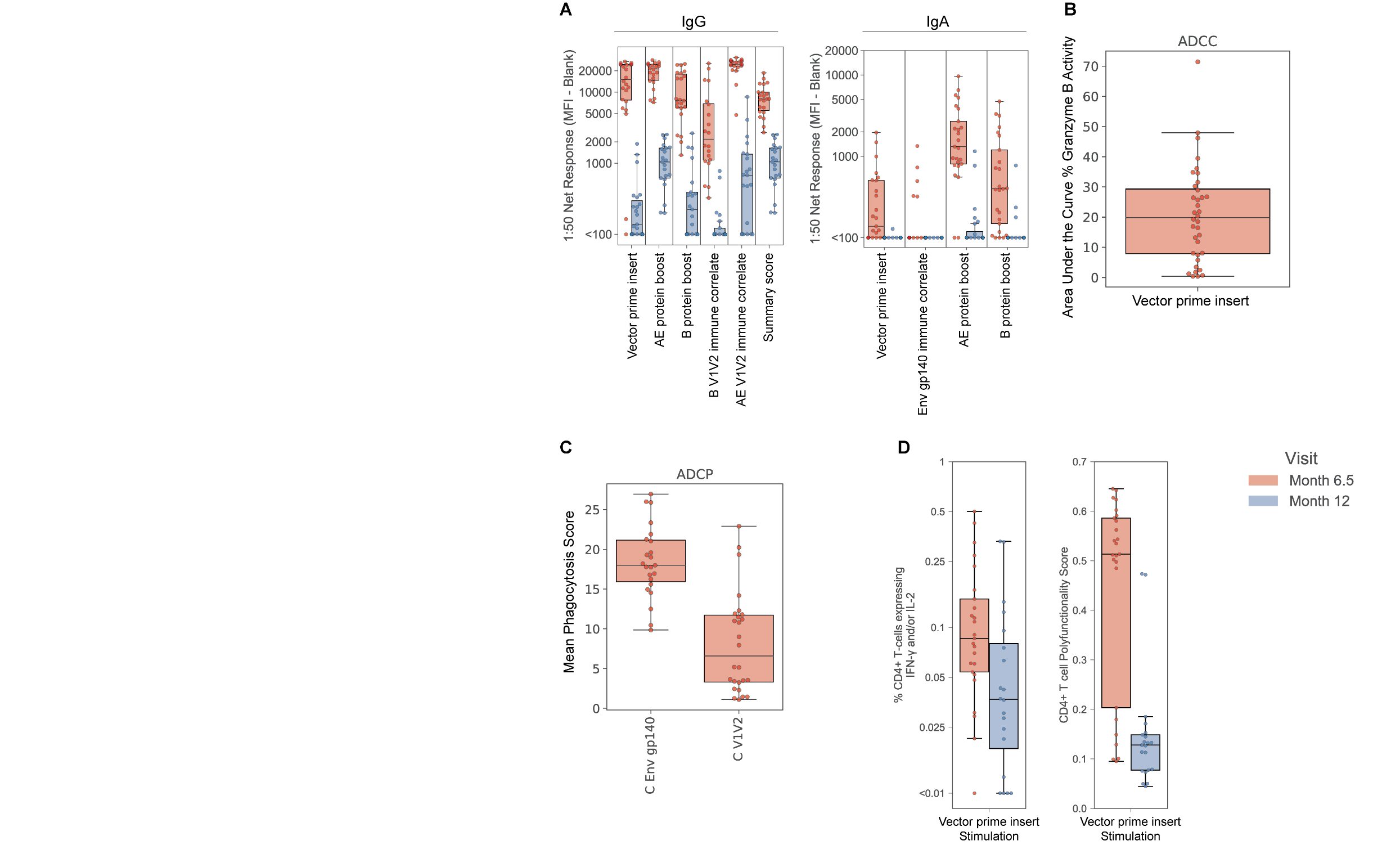


**S1 Fig.** Adaptive immune responses in HVTN 097 vaccine recipients used for assessing associations with innate responses. All adaptive immune responses were previously reported in (*17*) and the subset of participants who enrolled in the innate sampling schedule are shown here for convenience. Responses at Month 6.5 are shown in red; responses at Month 12 are shown in blue. **A**) Binding antibody responses. IgG (left panel) and IgA (right panel) binding antibody levels to HIV Env proteins as measured in a multiplex assay. The summary score is the area under the magnitude-breadth curve (*66*). **B**) Antibody-dependent cellular cytotoxicity responses were evaluated against AE 92TH023_gD-neg gp120–coated target cells using peripheral blood mononuclear cells from one normal healthy HIV-seronegative donor as the source of effector cells. The y-axis is the area under the curve (AUC) and represents the response magnitude as detected by release of granzyme B into target cells by flow cytometry. **C**) Antibody-dependent cellular phagocytosis responses. HIV-specific ADCP responses were measured by covalently binding HIV-1 antigens to fluorescent beads and incubating the beads in participant serum to enable formation of immune complexes. The complexes were then incubated with THP-1 monocyte-like cells, after which cell fluorescence was detected by flow cytometry. **D**) HIV-1 Env-specific CD4+ T-cell responses. (Left panel) CD4+ T cell responses as measured in the intracellular cytokine staining (ICS) assay, corresponding to the frequency of CD4+ T cells expressing IL-2 and/or IFN-γ in response to *ex vivo* stimulation with 92TH023 Env overlapping peptide pools. (Right panel) CD4+ T cell polyfunctionality scores based on expression of IFN-γ, IL-2, TNF-α, IL-4 and CD154 in the ICS assay calculated using the COMPASS method (*7*). For all plots in **A**) through **D**), median lines are shown and boxes indicate the interquartile range (IQR) with whiskers indicating the two most extreme data points within 1.5 times the IQR. All antigen labels are explained further in Methods.
