# Supplementary material for: Innate immune signatures to a partially-efficacious HIV vaccine predict correlates of HIV-1 infection risk
Source: PLoS Pathog. 2021 Mar 15;17(3):e1009363. doi: 10.1371/journal.ppat.1009363 (PMC7959397; doi:10.1371/journal.ppat.1009363)
Supplement: S2 Fig — The 32 DEGs shared between Days 1 and 3 were input into NetworkAnalyst [64] to generate a protein-protein interaction (PPI) network as described in Materials and Methods. “GO Immune System Process” pathways that were significantly enriched (pV ≤ 0.05) for the resultant genes in the network (n = 187) were analyzed using the ClueGO plugin [69] in Cytoscape [66]. All other settings were used at default. The CluePedia plugin [70] was used to display genes from the original PPI network associated with each enriched GO function or with multiple enriched GO functions. (DOCX) [file ppat.1009363.s003.docx]

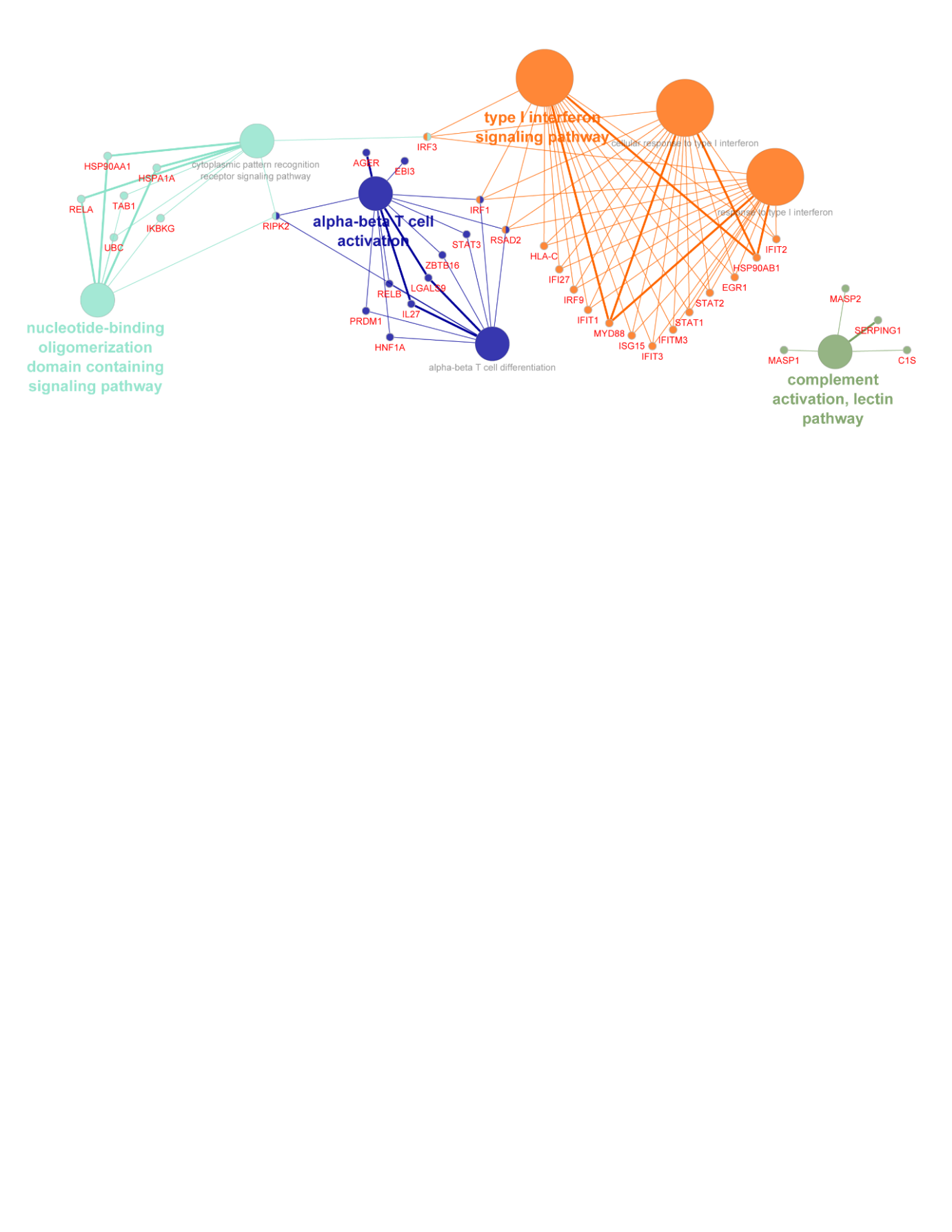


**S2 Fig.** Immune system functions in which the DEGs shared between Days 1 and 3 are involved. The 32 DEGs shared between Days 1 and 3 were input into NetworkAnalyst (*54*) to generate a protein-protein interaction (PPI) network as described in Methods. “GO Immune System Process” pathways that were significantly enriched (pV ≤ 0.05) for the resultant genes in the network (n=187) were analyzed using the ClueGO plugin (*67*) in Cytoscape (*56*). All other settings were used at default. The CluePedia plugin (*68*) was used to display genes from the original PPI network associated with each enriched GO function or with multiple enriched GO functions.
