# Supplementary material for: Innate immune signatures to a partially-efficacious HIV vaccine predict correlates of HIV-1 infection risk
Source: PLoS Pathog. 2021 Mar 15;17(3):e1009363. doi: 10.1371/journal.ppat.1009363 (PMC7959397; doi:10.1371/journal.ppat.1009363)
Supplement: S3 Fig — The x-axis of each plot shows the trimmed mean of M-values (TMM)-normalized RNAseq counts and the y-axis shows the mean ratio of ddPCR data. (DOCX) [file ppat.1009363.s004.docx]

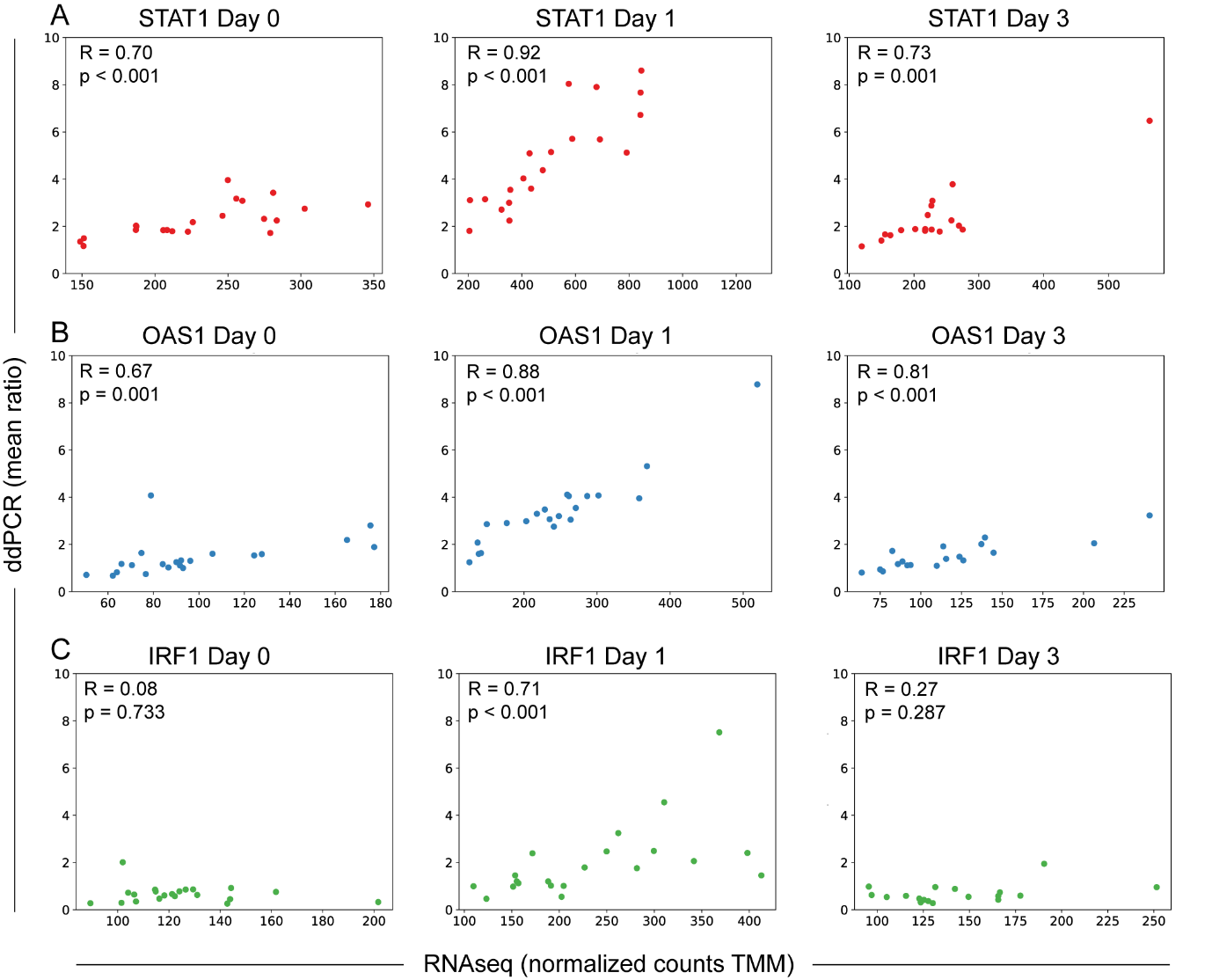


**S3 Fig.** Correlations of RNAseq data with ddPCR data for (A) *STAT1*, (B) *OAS1*, and (C) *IRF1* on Day 0 (left column), Day 1 (middle column), and Day 3 (right column). The x-axis of each plot shows the trimmed mean of M-values (TMM)-normalized RNAseq counts and the y-axis shows the mean ratio of ddPCR data.
