# Supplementary material for: Innate immune signatures to a partially-efficacious HIV vaccine predict correlates of HIV-1 infection risk
Source: PLoS Pathog. 2021 Mar 15;17(3):e1009363. doi: 10.1371/journal.ppat.1009363 (PMC7959397; doi:10.1371/journal.ppat.1009363)
Supplement: S4 Fig — A) Heatmap showing log(fold-change) in gene expression over baseline for the 783 DEGs (FDR≤0.2 and |FC|>1.5) in vaccine recipients at Days 1, 3, and 7 post-ALVAC vaccination. Each row represents one DEG. Each column represents one individual; the order of individuals is random, but consistent for each day. B) Table listing genes of interest from the UMAP clusters (interactive UMAP figure showing the Day 1, Day 3, and Day 7 log2 fold-change of each DEG at http://sieve.fredhutch.org/viz/VTN097). (DOCX) [file ppat.1009363.s005.docx]

B

A

**
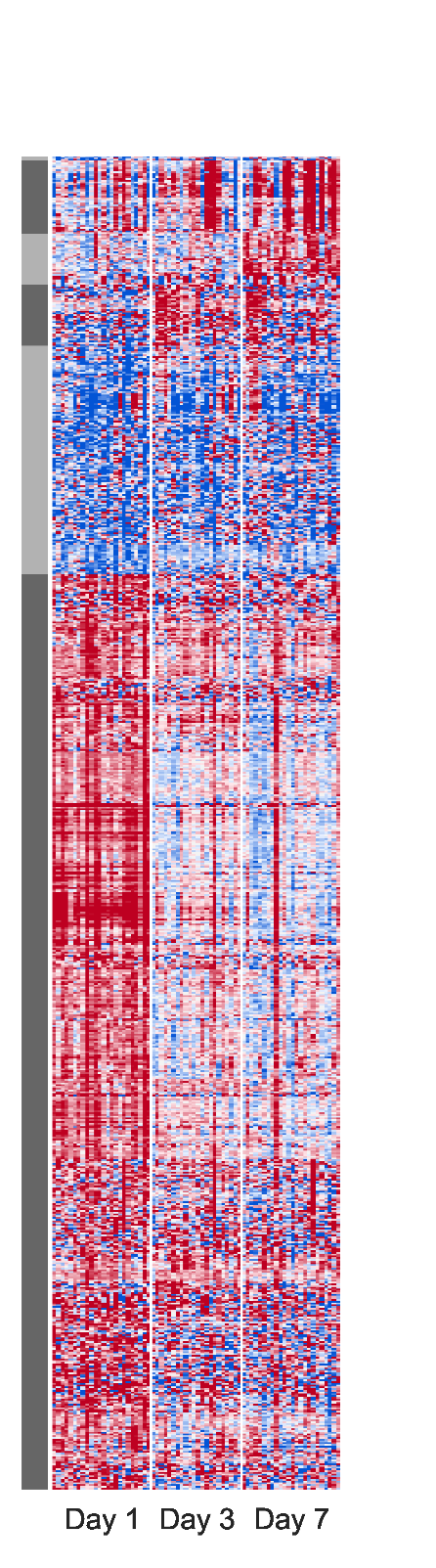
**

| **UMAP cluster** | **Pattern of expression** | **General description** | **Example DEGs** |
| --- | --- | --- | --- |
| I | Up Day 1, return to baseline by Day 7 | Anti-viral and pro-inflammatory | *OAS2*, *IFI44L*, *IFIT5*, *IRF7*, *DDX58* |
| II | Down Day 1, tended to stay down | Lymphocyte genes | *CD69*, *KLRC4*, *KIR3DL1*, *CXCR4*, *IFNG* |
| III | Up Day 3, tended to stay up | Mixed functions | *GPR132*, *ZNF557*, *ZNF8*, *ELMO3*, *AVIL* |
| IV | Up Day 7 | Immunoglobulin and B cell proliferation genes | *IGLV10-54*, *IGLV7-46*, *MK167*, *TOP2A*, *BIRC5* |
| V | Up Day 7 | Erythrocyte genes | *SLC4A1*, *EPB42*, *HBM*, *HBA1*, *HBB*, *HBD* |

**
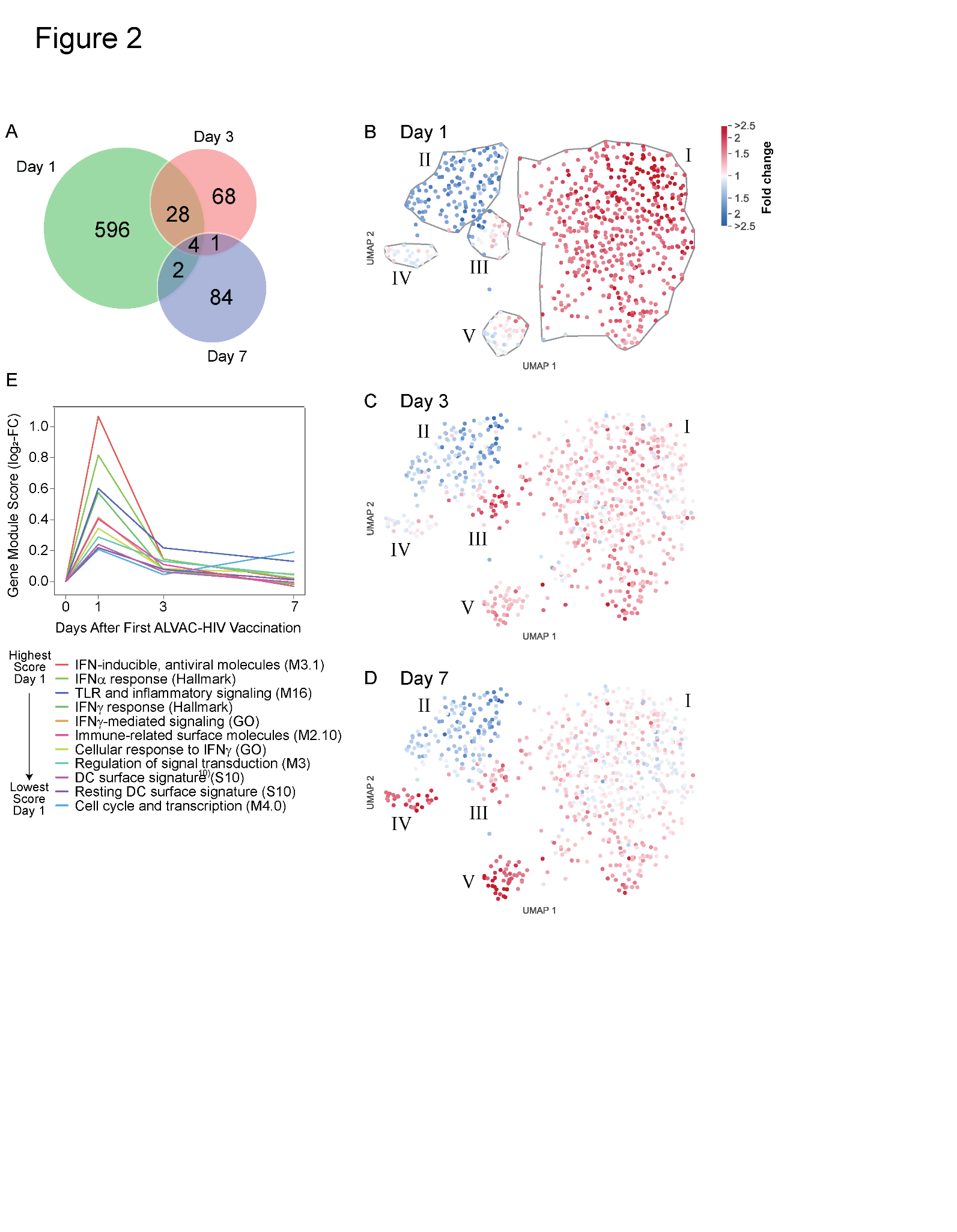
**

**S4 Fig.** **A)** Heatmap showing log(fold-change) in gene expression over baseline for the 783 DEGs (FDR≤0.2 and |FC|>1.5) in vaccine recipients at Days 1, 3, and 7 post-ALVAC vaccination. Each row represents one DEG. Each column represents one individual; the order of individuals is random, but consistent for each day. **B**) Table listing genes of interest from the UMAP clusters (interactive UMAP figure showing the Day 1, Day 3, and Day 7 log_2_ fold-change of each DEG at <http://sieve.fredhutch.org/viz/VTN097>).
