# Supplementary material for: Innate immune signatures to a partially-efficacious HIV vaccine predict correlates of HIV-1 infection risk
Source: PLoS Pathog. 2021 Mar 15;17(3):e1009363. doi: 10.1371/journal.ppat.1009363 (PMC7959397; doi:10.1371/journal.ppat.1009363)
Supplement: S5 Fig — Each line represents one participant. Cell types with significant enrichment or depletion compared to baseline (defined as an FWER < 0.05) are marked with a red asterisk on the appropriate day post-first ALVAC-HIV vaccination. The y-axis of each plot shows the proportion (percent) of the given cell type. CD16+ monocytes comprise the less-prevalent non-classical and intermediate monocyte populations; it is likely the day 3 increase is due to an increased proportion of intermediate CD16++ monocytes, similar to that observed after influenza vaccination [48]. (DOCX) [file ppat.1009363.s006.docx]

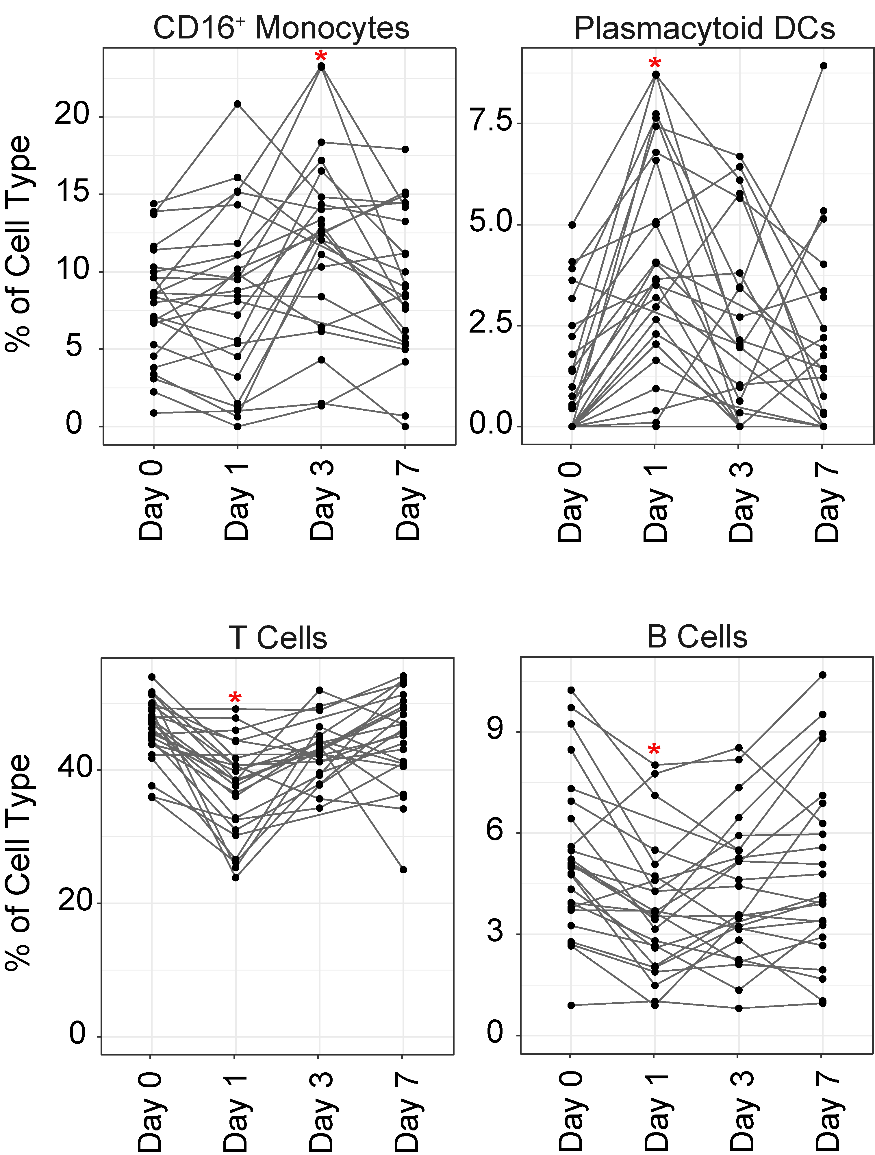


**S5 Fig.** Cellular enrichment analysis of RNA-seq data performed using immunoStates (22). Each line represents one participant. Cell types with significant enrichment or depletion compared to baseline (defined as an FWER < 0.05) are marked with a red asterisk on the appropriate day post-first ALVAC-HIV vaccination. The y-axis of each plot shows the proportion (percent) of the given cell type. CD16+ monocytes comprise the less-prevalent non-classical and intermediate monocyte populations; it is likely the day 3 increase is due to an increased proportion of intermediate CD16++ monocytes, similar to that observed after influenza vaccination (69).
