# Supplementary material for: Innate immune signatures to a partially-efficacious HIV vaccine predict correlates of HIV-1 infection risk
Source: PLoS Pathog. 2021 Mar 15;17(3):e1009363. doi: 10.1371/journal.ppat.1009363 (PMC7959397; doi:10.1371/journal.ppat.1009363)
Supplement: S6 Fig — R values of rank-based correlations are indicated on the upper right half of the grid and color-coded by the strength of the association; scatterplots are shown on the lower left half of the grid. (DOCX) [file ppat.1009363.s007.docx]

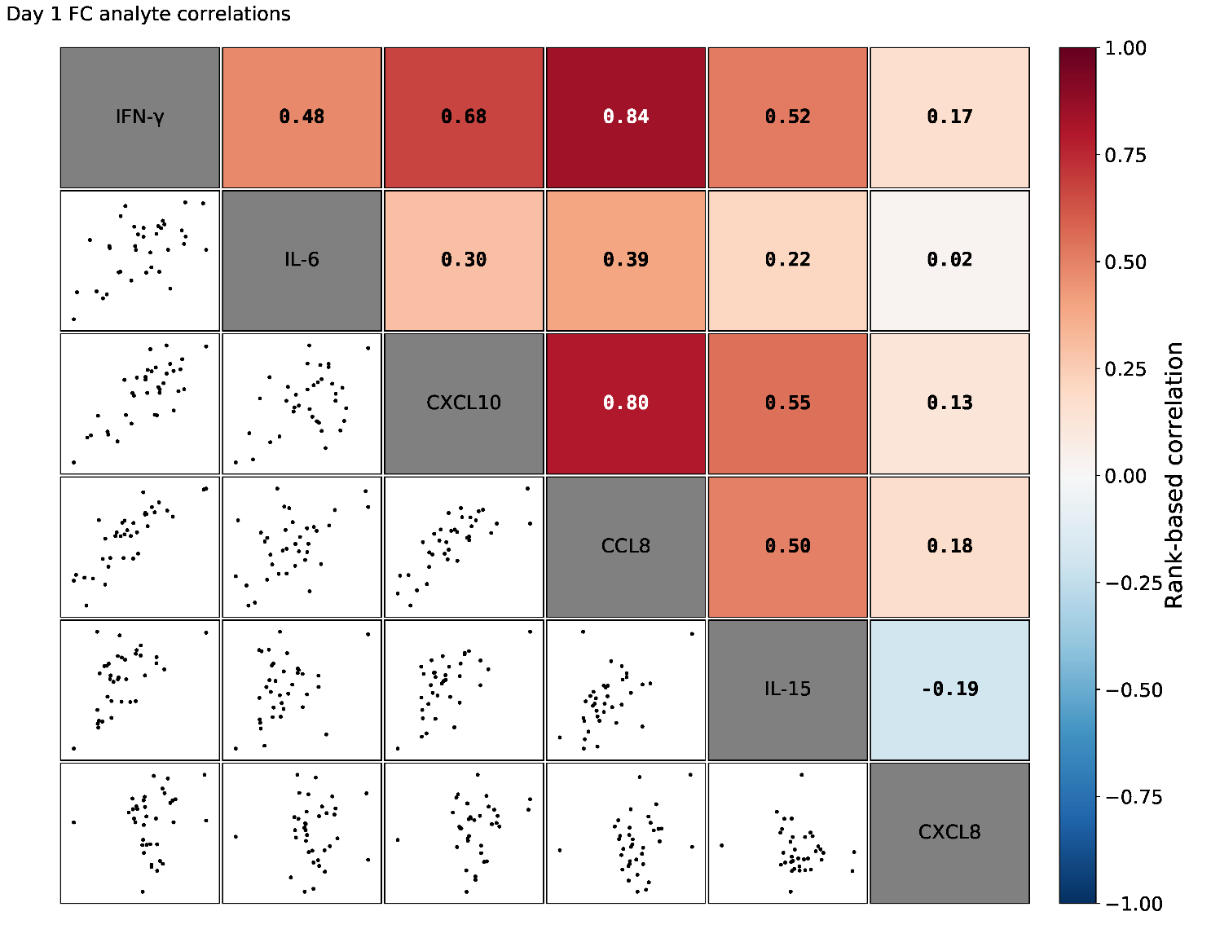


**S6 Fig.** Pairwise rank-based correlations among the fold-changes in concentration at Day 1 of the six serum factors that showed significant changes post-vaccination. R values of rank-based correlations are indicated on the upper right half of the grid and color-coded by the strength of the association; scatterplots are shown on the lower left half of the grid.
