# Supplementary material for: Innate immune signatures to a partially-efficacious HIV vaccine predict correlates of HIV-1 infection risk
Source: PLoS Pathog. 2021 Mar 15;17(3):e1009363. doi: 10.1371/journal.ppat.1009363 (PMC7959397; doi:10.1371/journal.ppat.1009363)
Supplement: S7 Fig — A) Heatmap showing log (fold-change) over baseline in placebo recipients for the 783 DEGs (FDR≤0.2 and |FC|>1.5) identified in vaccine recipients at Days 1, 3, and 7 post-ALVAC vaccination. Each row represents one DEG. Each column represents one individual; the order of individuals is random, but consistent for each day. Only placebo recipients (n = 4) are shown. B) Fold-change in placebo recipients (n = 7) over pre-vaccination level of the six serum factors that showed significant induction or repression after ALVAC-HIV vaccination. Serum cytokine concentrations were measured using the multiplexed MesoScale Discovery platform. Boxes extend through the interquartile range (IQR), with whiskers extending to the lowest and highest points within 1.5 times the IQR. No factors showed significant fold-changes in concentration relative to baseline (FWER-p <0.05, Wilcoxon signed-rank test). (DOCX) [file ppat.1009363.s008.docx]

**
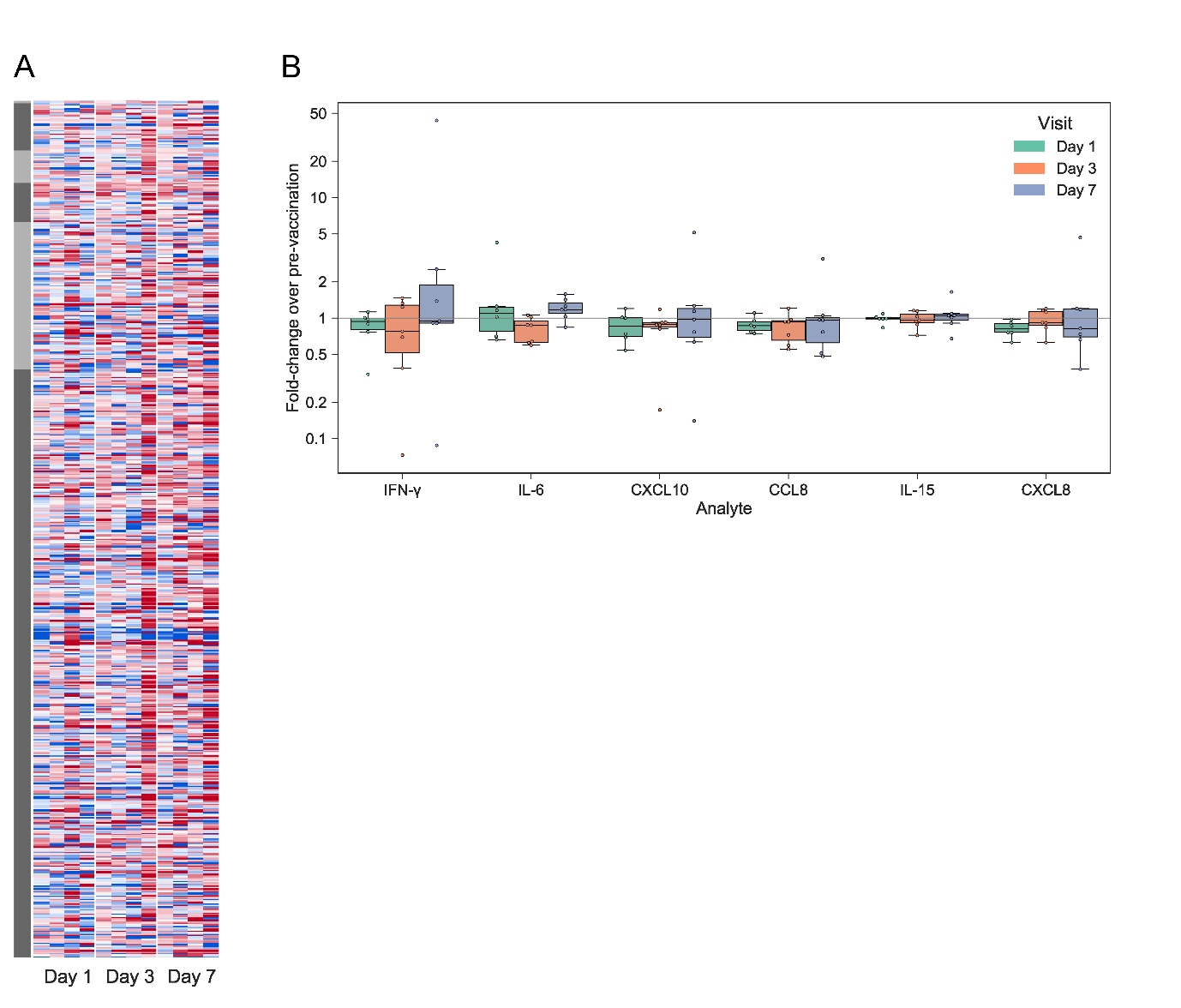
**

**S7 Fig. A)** Heatmap showing log (fold-change) over baseline in placebo recipients for the 783 DEGs (FDR≤0.2 and |FC|>1.5) identified in vaccine recipients at Days 1, 3, and 7 post-ALVAC vaccination. Each row represents one DEG. Each column represents one individual; the order of individuals is random, but consistent for each day. Only placebo recipients (n=4) are shown. **B**) Fold-change in placebo recipients (n=7) over pre-vaccination level of the six serum factors that showed significant induction or repression after ALVAC-HIV vaccination. Serum cytokine concentrations were measured using the multiplexed MesoScale Discovery platform. Boxes extend through the interquartile range (IQR), with whiskers extending to the lowest and highest points within 1.5 times the IQR. No factors showed significant fold-changes in concentration relative to baseline (FWER-p <0.05, Wilcoxon signed-rank test).
