# Supplementary material for: Innate immune signatures to a partially-efficacious HIV vaccine predict correlates of HIV-1 infection risk
Source: PLoS Pathog. 2021 Mar 15;17(3):e1009363. doi: 10.1371/journal.ppat.1009363 (PMC7959397; doi:10.1371/journal.ppat.1009363)
Supplement: S8 Fig — A) Median log2-reads at baseline (with 95% CI) of the 11 transcriptional modules activated at Day 1, for participants who received the tetanus vaccine (orange) and participants who did not receive the tetanus vaccine (green). B) Serum concentrations (pg/ml) at baseline for participants who received the tetanus vaccine (orange, including 7 who received placebo and 22 who received ALVAC-HIV) and participants who did not receive the tetanus vaccine (green) for the 6 cytokines with significant changes at Day 1 post-vaccination. Boxes indicate the interquartile range (IQR), with whiskers indicating the two most extreme data points within 1.5 times the IQR. (DOCX) [file ppat.1009363.s009.docx]

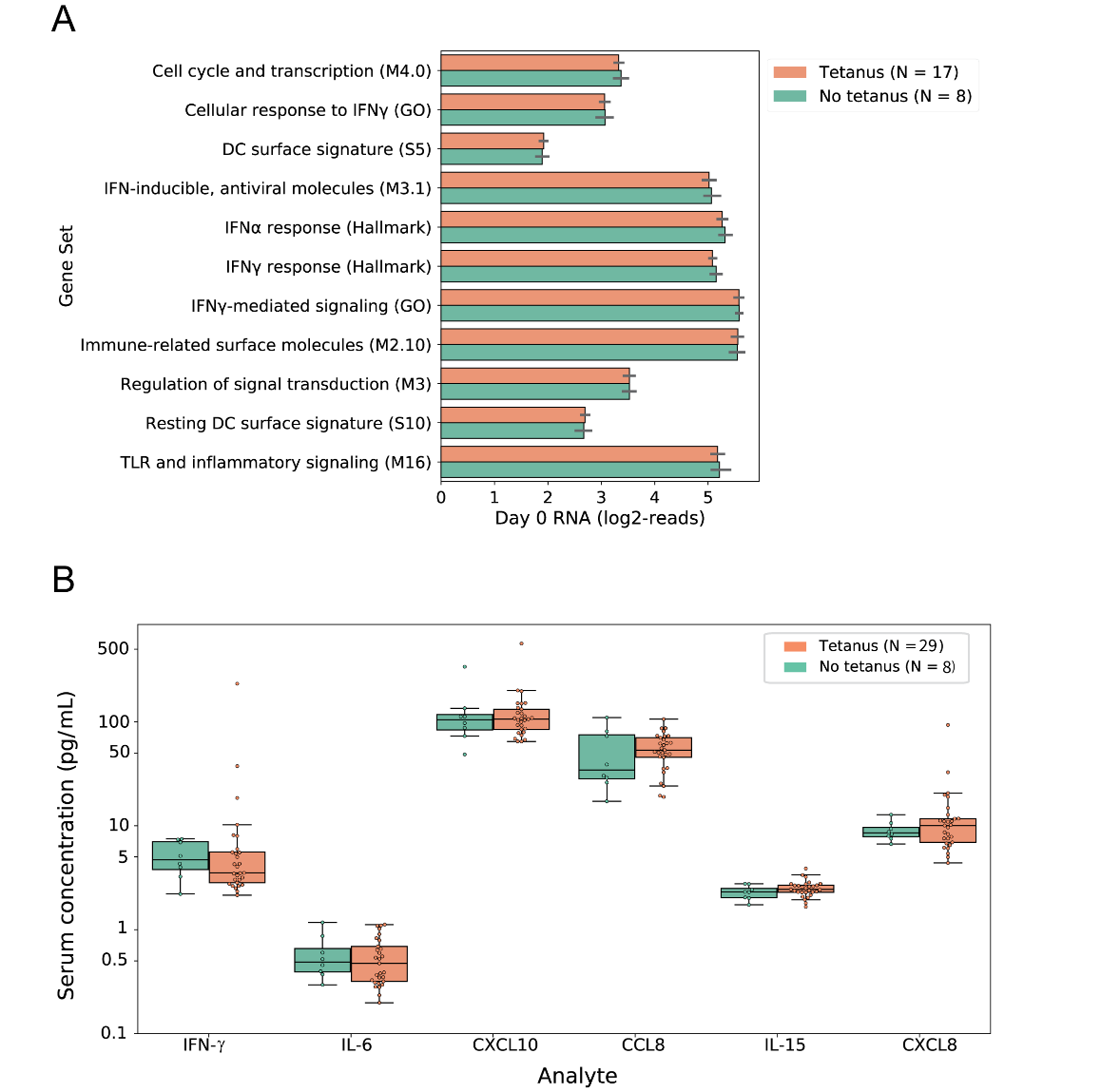


**S8 Fig**. Similar baseline profiles for participants who did vs did not receive the tetanus vaccine 1 month before the HIV vaccine regimen. **A**) Median log_2_-reads at baseline (with 95% CI) of the 11 transcriptional modules activated at Day 1, for participants who received the tetanus vaccine (orange) and participants who did not receive the tetanus vaccine (green). **B**) Serum concentrations (pg/ml) at baseline for participants who received the tetanus vaccine (orange, including 7 who received placebo and 22 who received ALVAC-HIV) and participants who did not receive the tetanus vaccine (green) for the 6 cytokines with significant changes at Day 1 post-vaccination. Boxes indicate the interquartile range (IQR), with whiskers indicating the two most extreme data points within 1.5 times the IQR.
