# Supplementary material for: Innate immune signatures to a partially-efficacious HIV vaccine predict correlates of HIV-1 infection risk
Source: PLoS Pathog. 2021 Mar 15;17(3):e1009363. doi: 10.1371/journal.ppat.1009363 (PMC7959397; doi:10.1371/journal.ppat.1009363)
Supplement: S9 Fig — A-B) Heatmaps representing the strength of rank-based correlations of fold-change in gene module scores with Months 6.5 and 12 IgG (A) and IgA (B) binding antibody responses. HIV-1 Env antigens are shown in columns (V1V2 IgG “immune correlate” = inverse correlates of risk in RV144; Env gp140 IgA “immune correlate” = direct correlate of risk in RV144). For detailed antigen names, see S1 Text. FDR-q<0.2 (+) or <0.1 (++). (DOCX) [file ppat.1009363.s010.docx]

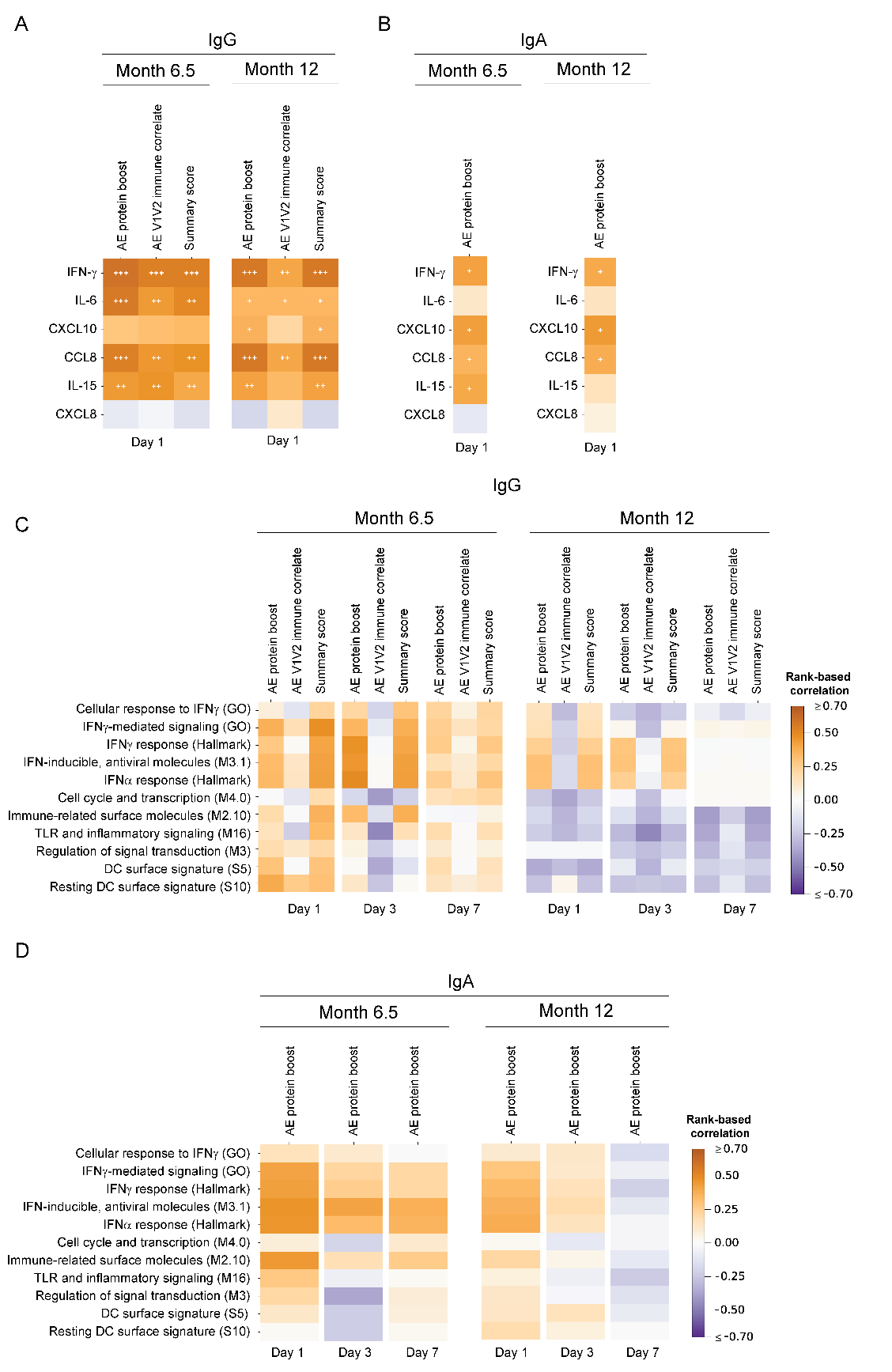


**S9 Fig.** Few associations between early changes in gene expression and Env-specific binding antibody responses. A-B) Heatmaps representing the strength of rank-based correlations of fold-change in gene module scores with Months 6.5 and 12 IgG (A) and IgA (B) binding antibody responses. HIV-1 Env antigens are shown in columns (V1V2 IgG “immune correlate” = inverse correlates of risk in RV144; Env gp140 IgA “immune correlate” = direct correlate of risk in RV144). For detailed antigen names, see Supplementary Methods. FDR-q<0.2 (+) or <0.1 (++).
