# Supplementary material for: Innate immune signatures to a partially-efficacious HIV vaccine predict correlates of HIV-1 infection risk
Source: PLoS Pathog. 2021 Mar 15;17(3):e1009363. doi: 10.1371/journal.ppat.1009363 (PMC7959397; doi:10.1371/journal.ppat.1009363)
Supplement: S1 Table — (DOCX) [file ppat.1009363.s011.docx]

## **S1 Table.** Demographic data from all HVTN 097 participants and from the subset of participants for whom PBMC transcriptional profiling was performed (median and IQR)

|  | All HVTN 097 participants (n=100) | Participants selected for PBMC transcriptional profiling (n=25) |
| --- | --- | --- |
| Age (yrs) | 22.0 (20.2, 25.3) | 23.5 (20.9, 26.4) |
| BMI | 21.7 (19.4, 26.7) | 20.9 (20.3, 26.3) |
| Female (%) | 49 | 52 |
| Height (cm) | 166.0 (159.8, 171.0) | 166.0 (161.0, 169.0) |
| Weight (kg) | 60.5 (55.8, 72.0) | 60.0 (56.0, 72.0) |
