# Supplementary material for: Innate immune signatures to a partially-efficacious HIV vaccine predict correlates of HIV-1 infection risk
Source: PLoS Pathog. 2021 Mar 15;17(3):e1009363. doi: 10.1371/journal.ppat.1009363 (PMC7959397; doi:10.1371/journal.ppat.1009363)
Supplement: S2 Table — (DOCX) [file ppat.1009363.s012.docx]

**S2 Table.** Numbers of differentially expressed genes relative to baseline on Days 1, 3, and 7 post-first ALVAC-HIV vaccination.
