# Supplementary material for: Innate immune signatures to a partially-efficacious HIV vaccine predict correlates of HIV-1 infection risk
Source: PLoS Pathog. 2021 Mar 15;17(3):e1009363. doi: 10.1371/journal.ppat.1009363 (PMC7959397; doi:10.1371/journal.ppat.1009363)
Supplement: S4 Table — (DOCX) [file ppat.1009363.s014.docx]

## **S4 Table.** References for modular transcriptional Gene Set Enrichment (GSEA)
